# Investigation of the relationship between inflammation and microbiota in the intestinal tissue of female and male rats fed with fructose: Modulatory role of metformin

**DOI:** 10.1007/s40199-024-00521-2

**Published:** 2024-06-17

**Authors:** Azimet Yalçın Buğdaycı, Saadet Özen Akarca Dizakar, Mürşide Ayşe Demirel, Suna Ömeroğlu, Fatma Akar, Mecit Orhan Uludağ

**Affiliations:** 1 Faculty of Pharmacy, Department of Pharmacology, Gazi University, Ankara, Turkey; 2 Faculty of Medicine, Department of Histology and Embryology, Bakırçay University, İzmir, Turkey; 3 Faculty of Pharmacy, Department of Basic Pharmaceutical Sciences, Gazi University, Ankara, Turkey; 4 Faculty of Medicine, Department of Histology and Embryology, Gazi University, Ankara, Turkey; 5 Faculty of Pharmacy, Department of Clinical Pharmacy, Near East University, TRNC, Lefkosa, Turkey

**Keywords:** High-fructose diet, Gut microbiota, Metformin, Metabolic syndrome, Inflammation, Gut permeability

## Abstract

**Background:**

It has been reported that High-Fructose (HF) consumption, considered one of the etiological factors of Metabolic Syndrome (MetS), causes changes in the gut microbiota and metabolic disorders. There is limited knowledge on the effects of metformin in HF-induced intestinal irregularities in male and female rats with MetS.

**Objectives:**

In this study, we investigated the sex-dependent effects of metformin treatment on the gut microbiota, intestinal Tight Junction (TJ) proteins, and inflammation parameters in HF-induced MetS.

**Methods:**

Fructose was given to the male and female rats as a 20% solution in drinking water for 15 weeks. Metformin (200 mg/kg) was administered by gastric tube once a day during the final seven weeks. Biochemical, histopathological, immunohistochemical, and bioinformatics analyses were performed. Differences were considered statistically significant at *p* < 0.05.

**Results:**

The metformin treatment in fructose-fed rats promoted glucose, insulin, Homeostasis Model Assessment of Insulin Resistance Index (HOMA-IR), and Triglyceride (TG) values in both sexes. The inflammation score was significantly decreased with metformin treatment in fructose-fed male and female rats (*p* < 0.05). Moreover, metformin treatment significantly decreased Interleukin-1 Beta (IL-1β) and Tumor Necrosis Factor-Alpha (TNF-α) in ileum tissue from fructose-fed males (*p* < 0.05). Intestinal immunoreactivity of Occludin and Claudin-1 was increased with metformin treatment in fructose-fed female rats. HF and metformin treatment changed the gut microbial composition. *Firmicutes/Bacteroidetes* (F/B) ratio increased with HF in females. In the disease group, *Bifidobacterium pseudolongum*; in the treatment group, *Lactobacillus helveticus* and *Lactobacillus reuteri* are the prominent species in both sexes. When the male and female groups were compared, *Akkermansia muciniphila* was prominent in the male treatment group.

**Conclusion:**

In conclusion, metformin treatment promoted biochemical parameters in both sexes of fructose-fed rats. Metformin showed a sex-dependent effect on inflammation parameters, permeability factors, and gut microbiota. Metformin has partly modulatory effects on fructose-induced intestinal changes.

**Graphical Abstract:**

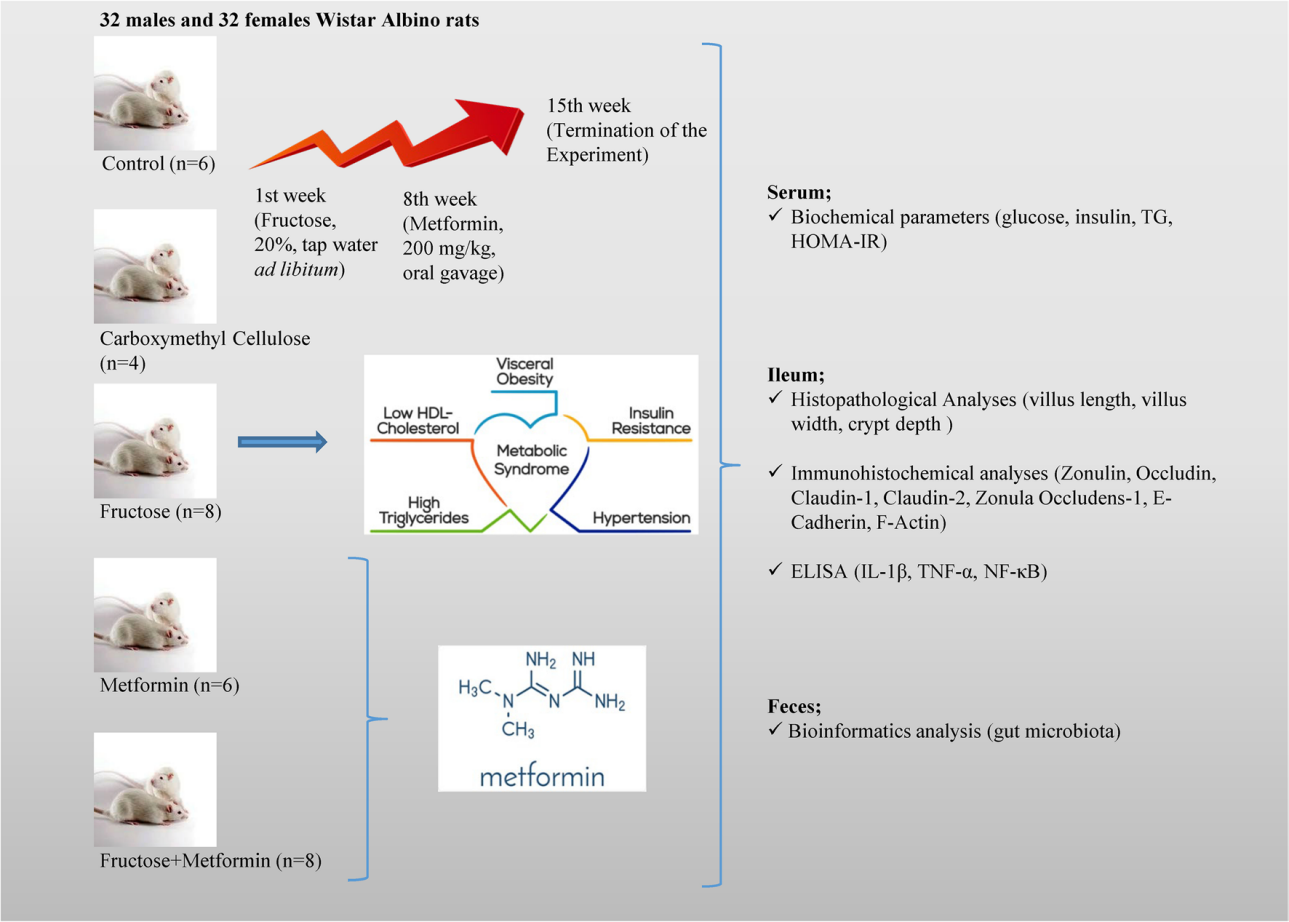

**Supplementary Information:**

The online version contains supplementary material available at 10.1007/s40199-024-00521-2.

## Introduction

MetS, caused by the imbalance between calorie intake and energy expenditure, is also a pathophysiological condition affected by other factors such as the individual's genetic/epigenetic structure, sedentary lifestyle, nutrition habits, and the composition of the gut microbiota [1]. Studies show a parallelism between the increase in fructose consumption and the characteristics of MetS [2]. Fructose-induced MetS has been shown to be closely associated with inflammation in the intestine, characterized by increased concentration of pro-inflammatory cytokines and activation of the inflammatory signaling pathway [3]. It has been shown that chronic fructose intake can also lead to a decrease in the levels of TJ proteins in the small intestine [4]. With the weakening of the mucosal barrier, the increase in the passage of antigens and other macromolecules from the external environment to the host causes local and/or systemic inflammation [5]. It has also been reported that a diet containing HF causes changes in gut microbiota [2].

Treatment of MetS includes diet and exercise along with pharmacological treatment of atherogenic dyslipidemia, hypertension, and hyperglycaemia [6]. Metformin, one of the drugs recommended for glucose intolerance in the first-line pharmacological treatment of MetS, is an orally used antihyperglycemic agent [7]. Metformin has also been shown to have beneficial effects in alleviating inflammation and inflammation-induced barrier breakdown [8]. High efficacy, low cost, minimal risk of hypoglycaemia when used as monotherapy, and the potential for some weight loss are the advantages of metformin, which is preferred as the first choice in treating many patients with MetS [9].

The mechanism of the antihyperglycemic effect of metformin, which has been used in treatment for a long time, is not fully understood [10]. Increasing evidence suggests that the gastrointestinal tract plays an important role in the antidiabetic effect of metformin. It has been previously highlighted that the lower small intestine is a site of action for metformin [11]. Intravenously infused metformin is known to accumulate more in the small intestine than in the liver or gallbladder. The accumulation of metformin in the small intestine may contribute to its glucose-lowering effect [12]. Also, studies in both animals and humans have suggested that metformin may show therapeutic effect by modulating gut microbiota [13–15], and modulation of the gut microbiota by metformin treatment may lead to improvement of metabolic parameters [16]. Although current studies have focused on this issue, the effect of metformin treatment on gut microbiota, the mechanism of action, and the metabolic pathways involved in treatment have not yet been clarified. In addition, there is not enough information about the effects of sex-specific differences on metformin-mediated changes in gut microbiota in fructose-induced MetS.

In this study, the modulatory role of metformin on the gut microbiota of male and female rats in which dietary HF induced MetS in the presence of intestinal TJ proteins and inflammation parameters was investigated for the first time. For this purpose, biochemical parameters, histopathological and immunohistochemical analyses in serum and ileum tissue, and bioinformatics analyses in cecal contents were performed to reveal sex-specific differences.

## Materials and methods

### Experimental animals

A total of 64 healthy Wistar Albino rats, 32 males and 32 females, were used in this study, which was approved by Gazi University Animal Experiments Local Ethics Committee with the decision numbered G.U.ET-18.034. Male and female rats weighing 75–85 g were admitted to Gazi University, Faculty of Pharmacy, Experimental Animal Care and Research Unit when they were three weeks old, and they were subjected to a one week adaptation and quarantine period. The experimental animals were kept in an environment where the temperature was 21-24ºC, and the humidity was 45–55% for a 12-h day/night time period. Rats were fed standard rat chow (protein: 23.0%, cellulose: 3.5%, NDF: 1.0%, fat: 5.5%, ash: 8.0%, lysine: 1.35%, methionine: 0.45%, cystine: 0.35%, Ca: 1.0%, P: 0.75%, Vitamin A: 25 000 IU/kg, Vitamin D: 4000 IU/kg, Vitamin E: 80 mg/kg, choline: 1700 mg/kg, iron: 100 mg/kg, copper: 15 mg/kg, zinc: 120 mg/kg, manganese: 140 mg/kg, iodine: 1.2 mg/kg) and tap water ad libitum for 15 weeks. The body weights of all animals were measured and recorded once a week during the experimental protocol.

### Design of experimental groups and MetS

Experimental animals of both sexes were randomly divided into five groups: Control (C, n = 6), Carboxymethyl cellulose (CMC, n = 4), Metformin (M, n = 6), Fructose (F, n = 8), and Fructose + Metformin (FM, n = 8) after one week adaptation period. In order to design the MetS experimental model, 20% fructose was added to the drinking water of F and FM groups starting from the first week until the end of the experimental period. In addition, starting from the eight week until the end of the 15th week, the C groups received saline (1 ml, *per os*); M and FM groups received 200 mg/kg metformin [17] dissolved in carboxymethyl cellulose (1 ml, *per os*); and CMC groups received 0.5% carboxymethyl cellulose (1 ml, *per os*). In the eighth week of the experimental procedure, glucose and TG levels were measured in blood samples taken from the tail vein, confirming that the disease model had been created, and the treatment protocol was started.

### Termination of the experiment

At the end of the 15th week, the animals were sacrificed by intracardiac blood sampling under general anesthesia with xylazine (8 mg/kg) and ketamine (75 mg/kg) administered intraperitoneally. Ileum tissue was dissected for biochemical and histopathological analyses. Tissue samples were stored at -80 °C (Sanyo-MDF-U5186S) until the analyses were performed. Feces samples were taken from the cecum for microbiota analysis, and then the samples were delivered under appropriate conditions to the company from which the service was purchased.

### Biochemical parameters

#### Biochemistry analysis in serum by ELISA method

The obtained blood sample was centrifuged at 3000 rpm for 15 min (ZIP IQ LW Scientific), and serums were separated and stored in a -80 °C cooler (Sanyo-MDF-U5186S) until analyses were performed. In serum samples, glucose (BioVision, USA), insulin (Cusabio, Wuhan, China), and TG (BioVision, USA) levels were measured using species-specific Enzyme-Linked Immunosorbent Assay (ELISA) kits according to the manufacturer's instructions. These kits are for the quantitative determination of rat glucose, insulin, and TG concentrations. HOMA-IR calculations were performed to determine insulin resistance (Homa index = insulin x glucose/22.5).

#### Protein analysis in ileum tissue by ELISA method

The distal part of the ileum tissues of the animals was homogenized on ice using a homogeniser (SONICS Vibracell) by adding Phosphate Buffered Saline (PBS) (pH: 7.4) for spectrophotometric analyses. The homogenates were centrifuged at 2000–3000 rpm for 20 min at four °C (PLU NF800R). IL-1β (ScienCell, Shanghai, China), TNF-α (ScienCell, Shanghai, China), and nuclear factor kappa B (NF-κB) (Cusabio, Wuhan, China) levels in the supernatants were measured using species-specific ELISA kits according to the manufacturer's instructions**.** These kits are for the quantitative determination of rat glucose, insulin, and TG concentrations.

### Histopathological analyses

The proximal part of the ileum tissues was washed after fixation in 10% neutral formaldehyde. Washed ileum samples were dehydrated by passing through an increasing series of alcohol. After clearing with xylol, it was embedded in paraffin. Then four µm thick sections were stained with Hematoxylin–Eosin.

All slides were obtained under a computer-assisted light microscope (Leica DM4000B, Germany) and evaluated with Leica LAS V4.9. Randomized ten villi and crypts were selected from each slide, and villus length, villus width, and crypt depth were evaluated histomorphometrically using Image J software (National Institutes of Health, USA) [18].

Histopathological scoring of ileum tissues was calculated according to the total value of inflammation (0:absent – 4:severe), transmural inflammation (0:absent – 3:severe), crypt and epithelial damage (0:absent – 4:severe), tissue regeneration (0:regenerated or normal 4:no repair) parameters [19]. All scores were evaluated by two different researchers who were blinded for the sample.

### Immunohistochemical analyses

After deparaffinization, the ileum sections were incubated in citrate buffer (pH: 6.0) (Lab Vision, Thermo Scientific, Fremont) and 3% hydrogen peroxide (Lab Vision, Thermo Scientific, Fremont). After washing with PBS (Thermo, AP-9009–10, UK) (pH: 7.4), Ultra V block (Lab Vision, Thermo Scientific, Fremont) was applied to prevent non-specific binding. Following the blocking stage, tissue sections were incubated with Zonulin (bs-1808R, Bioss), Occludin (bs-1495R, Bioss), Claudin-1 (bs-1428R, Bioss), Claudin-2 (bs-7125R, Bioss), Zonula Occludens-1 (ZO-1) (bs-1329R, Bioss), E-Cadherin (bs-10009R, Bioss), and F-Actin (bs-1571R, Bioss) primer antibodies (1:200 dilution) overnight. Then, the sections were incubated with secondary antibodies (Lab Vision, Thermo Scientific, Fremont) for 10 min. The reaction product was revealed by streptavidin peroxidase complex (Lab Vision, Thermo Scientific, Fremont) with Diaminobenzidine (DAB). The percentage of immunopositivity of primer antibodies was determined in each area using the ImageJ (Java-based software program, National Institutes of Health).

### Bioinformatics Analysis in Feces by 16S Ribosomal Ribonucleic Acid (rRNA) Gene Sequencing

#### Deoxyribonucleic Acid (DNA) extraction

Collected feces samples were weighed 25–50 mg and transferred to tubes containing sterile Tris–EDTA buffers. The solution obtained by rapid mixing in a cold environment for about one hour was then centrifuged for 15 min at 13 000 g to remove the supernatant and obtain a pellet.

After centrifugation, the supernatant was discarded, and extraction continued with the pellet. EurX GeneMATRIX Tissue & Bacterial DNA Purification Kit (Cat No: E3551, Gdansk Poland) was used to extract DNA from the pellet. The pellet was applied to 250 µl of Stool Lysis solution and 25 µl of Proteinase K solution and incubated at 56 °C for 60 min. After adding 200 µl Lysis solution, the tubes were vortexed for 15 s and incubated at 70 °C for 10 min. Then 250 µl 99% cold ethanol was added to the tubes and vortexed for 15 s. The whole volume inside the microcentrifuge tube was immediately transferred to the column. The column was transferred to a new tube after centrifugation at 8000 g × 1 min. Then, after washing according to the kit protocol, 100 µl of Elution solution was added, and after 2 min of incubation, genomic DNA was obtained by centrifugation at 8000 g × 1 min. DNA concentrations were determined using the Qubit 2.0 Fluorometer, and DNA samples were stored at -20 °C until use.

#### Sequencing analysis

From the isolated DNA samples, the V3-V4 region of the 16S rRNA gene hypervariable regions was amplified with primers 341F (CCTAYGGGRBGCASCAG) and 806R (GGACTACNNGGGTATCTAAT). Then 12.5 µL Kapa HiFi HotStart Mastermix (Kapa Biosystems, USA Cat. No: KK2601), (10 µM) 0.5 µL 341f primer, (10 µM) 0.5 µL 806r primer, and DNA sample adjusted to a concentration of 20 ng were added to the reaction carried out at a total of 25 µL. The remaining amount was completed with nuclease-free water. The reaction was initiated at 95 °C for three minutes; 28 cycles were carried out at 95 °C for 30 s, at 55 °C for 30 s; and at 72 °C for 45 s and completed with 10 min binding at 72 °C. Ampure XP beads (Beckman Cat No: A63880) were used to clean the primers after PCR. Then, barcode and index primers were added to both ends of the PCR products with Illumina Nextera XT Index kit v2 Set A (Cat No: FC-131–2001). After Nextera, the samples whose primers were cleaned with Ampure were diluted to a concentration of 3.9 ng/µL. Then, 5 µL was taken from each sample, and a sample pool was created. Sequencing was performed on the Illumina Miseq platform using a MiseqReagent V2 kit (Illumina, USA Cat No: MS-102–2002), and 2 × 300 bp paired-end reads were obtained. The sequencing step was done simultaneously. In other words, library preparation and sequencing of all samples on the device were carried out cumulatively.

#### Bioinformatics analysis

Pair-end Illumina reads (2 × 250) were imported to the qiime2 environment [20]. All of the samples had more than 100X sequence depth, and no samples were removed from the study. Bases with a low phred score (< Q30) of reads [21] applied through quality clipping, chimera detection, and the Qiime2 Dada2 pipeline (via q2-dada2) were cut out. Amplicon Sequence Variants (ASV) generated by Dada2 were mapped to Silva 138 (https://www.arb-silva.de/documentation/release-138/) database [22, 23]. The Phyloseq [24] object was created from qiime2 artifact files in the R 4.1 environment [25]. Alpha diversity assessment, used to evaluate the diversity of related taxonomic units in a sample, was interpreted using two different indices, including Chao1 and Shannon. P values between groups were calculated with the Kruskal–Wallis test [26]. Beta diversity analysis, used to assess taxonomic differences between individuals, was calculated based on Bray–Curtis [27, 28]. Specific differences between groups were determined by differential abundance analysis, Deseq2 R pack [29]. Linear Discriminant Analysis Effect Size (LEfSe) analysis was conducted between groups to show statistically significant taxonomies [30].

### Statistical analysis

The results obtained from the experiments were expressed as "mean ± standard error of the mean (SEM)". Differences between groups were evaluated using a one-way analysis of variance (ANOVA) and the Bonferroni test as post-hoc. Differences were considered statistically significant at p < 0.05. Student t-test was used to compare each parameter between the two groups. Statistical analyses were performed using a software package (GraphPad Prism version 5.01 for Windows: GraphPad Software, Inc., La Jolla, USA).

## Results

### Body weight changes of male and female rats

Body weight changes of male and female rats are shown in Table 1. When the weight gains of male and female rat groups were compared at the end of the experiment period, no statistically significant difference was found between the male rat groups. In female rats, the highest weight gain was observed in the F group, and the lowest weight gain was observed in the M group. When compared between sexes, it was observed that weight gain was higher in male rats compared to female rats (Table 1).

**Table 1:** Comparison of body weight changes of male and female rats (C, *n* = 6; CMC, *n* = 4; M, *n* = 6; F, *n* = 8; FM, *n* = 8). Intergroup analysis: ^#^Different from M group, one-way ANOVA, post-hoc Bonferroni test (*p* < 0.05). Cross-sex analysis: ^a^different from the same group of females, Student t-test (*p* < 0.05)

	Starting Body Weight (g)		Final Body Weight (g)		Weight Increase (g)	
Groups	Male	Female	Male	Female	Male	Female
C	144.2 ± 2.9	139.5 ± 4.4	519.5 ± 7.0^a^	298.0 ± 9.7	375.3 ± 7.1^a^	158.5 ± 10.0
CMC	144.8 ± 2.6	137.5 ± 6.5	482.5 ± 7.8^a^	298.8 ± 14.5	337.8 ± 8.2^a^	161.3 ± 15.1
M	145.5 ± 2.5	136.0 ± 6.5	502.5 ± 10.7^a^	278.3 ± 7.8	357.0 ± 12.2^a^	142.3 ± 6.5
F	143.3 ± 0.5	141.1 ± 4.9	507.9 ± 14.5^a^	324.1 ± 5.6^#^	364.6 ± 14.4^a^	183.0 ± 6.5^#^
FM	147.7 ± 5.0	138.1 ± 5.4	495.8 ± 19.5^a^	317.9 ± 5.6^#^	348.1 ± 21.1^a^	179.8 ± 5.1^#^

### Serum biochemistry findings of male and female rats

#### Serum glucose, insulin, HOMA-IR, and TG values

In male and female rats, serum glucose, insulin, HOMA-IR, and TG levels increased in the F group and decreased in the FM group (Table 2; Fig. S1a and b, Fig. S2a and b, Fig. S3a and b, Fig. S4a and b). Although a decreasing trend in glucose levels was observed in the FM group of male rats compared to the F group, it was not statistically significant (Table 2; Fig. S1a). When compared between sexes, serum glucose levels were found to be lower in male F and FM groups compared to the same female groups, and serum insulin level was lower in the male M group than in the female M group (Table 2). There was no statistically significant difference between the HOMA-IR values of male and female rat groups, and the TG level was lower in the male FM group than in the same female group (Table 2).

**Table 2:** Serum Glucose (mg/dL), Insulin (pmol/L), HOMA-IR, and TG (mg/dL) values of male and female rats (C, *n* = 6; CMC, *n* = 4; M, *n* = 6; F, *n* = 8; FM, *n* = 8). Intergroup analysis: ^*^Different from C group, ^+^different from CMC group, ^#^different from M group, ^&^different from FM group, one-way ANOVA, post-hoc Bonferroni test (*p* < 0.05). Cross-sex analysis: ^a^Different from the same group of females, Student t-test (*p* < 0.05)

	**C**	**CMC**	**M**	**F**	**FM**
Male					
Glucose	111.24 ± 3.6	118.96 ± 4.2	119.60 ± 3.0	132.33 ± 3.6^*a^	118.65 ± 3.5^a^
Insulin	248.96 ± 11.3	240.15 ± 13.9	239.57 ± 9.3^a^	429.68 ± 30.6^*+#&^	290.95 ± 13.1
HOMA-IR	9.91 ± 0.7	10.12 ± 0.5	10.20 ± 0.5	20.36 ± 1.8^*+#&^	12.31 ± 0.7
TG	146.24 ± 5.2	105.94 ± 4.9	105.94 ± 4.9	596.14 ± 30.5^*+#&^	279.06 ± 14.9^a^
Female					
Glucose	119.02 ± 3.7	117.78 ± 3.5	114.73 ± 3.9	147.03 ± 3.0^*+#&^	129.92 ± 3.4^#^
Insulin	256.40 ± 9.4	269.15 ± 9.0	279.27 ± 5.2	364.47 ± 20.3^*+#&^	293.27 ± 11.8
HOMA-IR	10.9 ± 0.7	11.27 ± 0.5	11.39 ± 0.4	19.01 ± 1.0^*+#&^	13.57 ± 0.7
TG	158.11 ± 9.1	114.91 ± 5.5	134.01 ± 9.4	602.74 ± 32.3^*+#&^	410.42 ± 16.6

### Inflammation markers measured in ileal tissue of male and female rats

It was found that IL-1β level was higher in the F group compared to the C group in both male and female rats (Table 3; Fig. S5a and b). It was observed that the IL-1β level decreased in the male FM group compared to the F group (Table 3; Fig. S5a). When male and female rats were compared, the IL-1β level of the male FM group was lower than that of the female FM group (Table 3). In male rats, the TNF-α level, which increased in the fructose group compared to the control group, decreased in the FM group (Table 3; Fig. S6a). In female rats, there was no statistically significant difference between the groups (Table 3; Fig. S6b). When compared between sexes, TNF-α levels were lower in males than females in the CMC group and higher in the F group (Table 3). When NF-κB levels were analyzed in male and female rats, no statistically significant difference was found between groups and sexes (Table 3; Fig. S7a and b).

**Table 3:** IL-1β (pg/mg), TNF-α (pg/mg), and NF-κB (pg/mg) values measured in the ileum tissue of male and female rats (C, *n* = 6; CMC, *n* = 4; M, *n* = 6; F, *n* = 8; FM, *n* = 8). Intergroup analysis: ^*^Different from C group, ^+^different from CMC group, ^#^different from M group, ^&^different from FM group, one-way ANOVA, post-hoc Bonferroni test (*p* < 0.05). Cross-sex analysis: ^a^Different from the same group of females, Student t-test (*p* < 0.05)

	C	CMC	M	F	FM
Male					
IL-1β	330.18 ± 24.8	317.82 ± 12.0	300.65 ± 36.0	444.56 ± 24.7^*+#&^	304.82 ± 22.5^a^
TNF-α	39.78 ± 1.5	37.41 ± 1.7^a^	45.61 ± 4.9	57.09 ± 1.4^*+&a^	43.66 ± 3.1
NF-κB	24.14 ± 6.1	33.42 ± 3.8	16.05 ± 3.9	36.47 ± 7.3	40.23 ± 8.4
Female					
IL-1β	356.9 ± 22.3	359.35 ± 22.1	299.35 ± 28.4^&^	520.36 ± 36.1^*#^	493.27 ± 38.2
TNF-α	48.37 ± 6.4	50.35 ± 2.6	57.14 ± 5.8	47.99 ± 2.4	55.93 ± 5.0
NF-κB	25.96 ± 14.2	14.74 ± 11.4	29.03 ± 8.2	24.60 ± 5.8	24.75 ± 2.9

### Histopathological analysis findings in ileum tissue of male and female rats

#### Histomorphometric measurements of ileum tissue of male and female rats

In male and female rats, the villus length of the F group was found to be lower than the Cgroup. On the other hand, villus length was higher in the FM group compared to the F group (Table 4; Fig. S8a and b). When male and female rats were compared, villus length was lower in males than females in the FM group but higher in the F group (Table 4).

**Table 4:** Villus length (µm), villus width (µm), and crypt depth (µm) measurements of ileum tissue of male and female rats (C, n = 6; CMC, n = 4; M, n = 6; F, n = 8; FM, n = 8). Intergroup analysis: ^*^Different from C group, ^+^different from CMC group, ^#^different from M group, ^β^different from F group,^&^different from FM group, one-way ANOVA, post-hoc Bonferroni test (p < 0.05). Cross-sex analysis: ^a^Different from the same group of females, Student t-test (p < 0.05)

	C	CMC	M	F	FM
Male					
Villus Length	254.31 ± 4.2	241.17 ± 3.1^a^	270.39 ± 9.7	215.23 ± 4.7^*#&a^	292.64 ± 5.8^*+a^
Villus Width	122.13 ± 3.5^a^	110.31 ± 5.1^#^	136.02 ± 3.1^a^	122.33 ± 2.4^a^	119.21 ± 3.9^#a^
Crypt Depth	191.48 ± 2.9	192.61 ± 7.0^a^	234.25 ± 3.1^*+β&a^	201.85 ± 2.9^a^	208.43 ± 5.5^a^
Female					
Villus Length	238.76 ± 10.7	221.53 ± 4.7	254.72 ± 11.9	186.99 ± 5.5^*#&^	327.80 ± 3.4^*+#^
Villus Width	106.85 ± 2.2	106.90 ± 3.9	91.03 ± 4.8	109.70 ± 3.2	104.35 ± 5.6
Crypt Depth	200.96 ± 5.4	125.91 ± 3.3^*# β&^	203.03 ± 5.7	158.99 ± 3.5^*#&^	187.51 ± 3.5

There was no statistically significant difference between the groups when the width of the villus was evaluated in male and female rats (Table 4; Fig. S9a and b). When compared between sexes, villus width was higher in males than females in all groups (Table 4).

The crypt depth was lower in the F group of female rats compared to the C and FM groups (Table 4; Fig. S10b). When compared between sexes, crypt depth was higher in males than females in the F and FM groups (Table 4).

#### Histopathological evaluation of inflammation of the ileum tissue of male and female rats

**Fig. 1:**
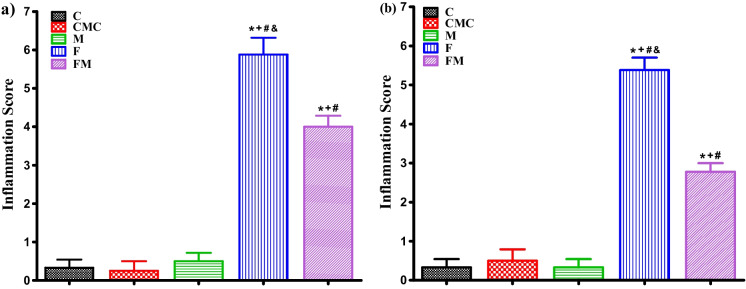
Inflammation scores of the ileum tissue of male (**a**) and female (**b**) rats (C, *n* = 6; CMC, *n* = 4; M, *n *= 6; F, *n* = 8; FM, *n* = 8). Intergroup analysis: ^*^Different from C group, ^+^different from CMC group, ^#^different from M group, ^&^different from FM group, one-way ANOVA, post-hoc Bonferroni test (*p* < 0.05)

In both male and female rats, the inflammation score was higher in the F group compared to the C group. In addition, the inflammation score was lower in the FM group compared to the F group in both sexes (Fig. 1a and b).

When male and female rats were compared, it was found that the inflammation score of the female FM group was lower than the male FM group. There is no statistically significant difference between other groups.

**Fig. 2:**
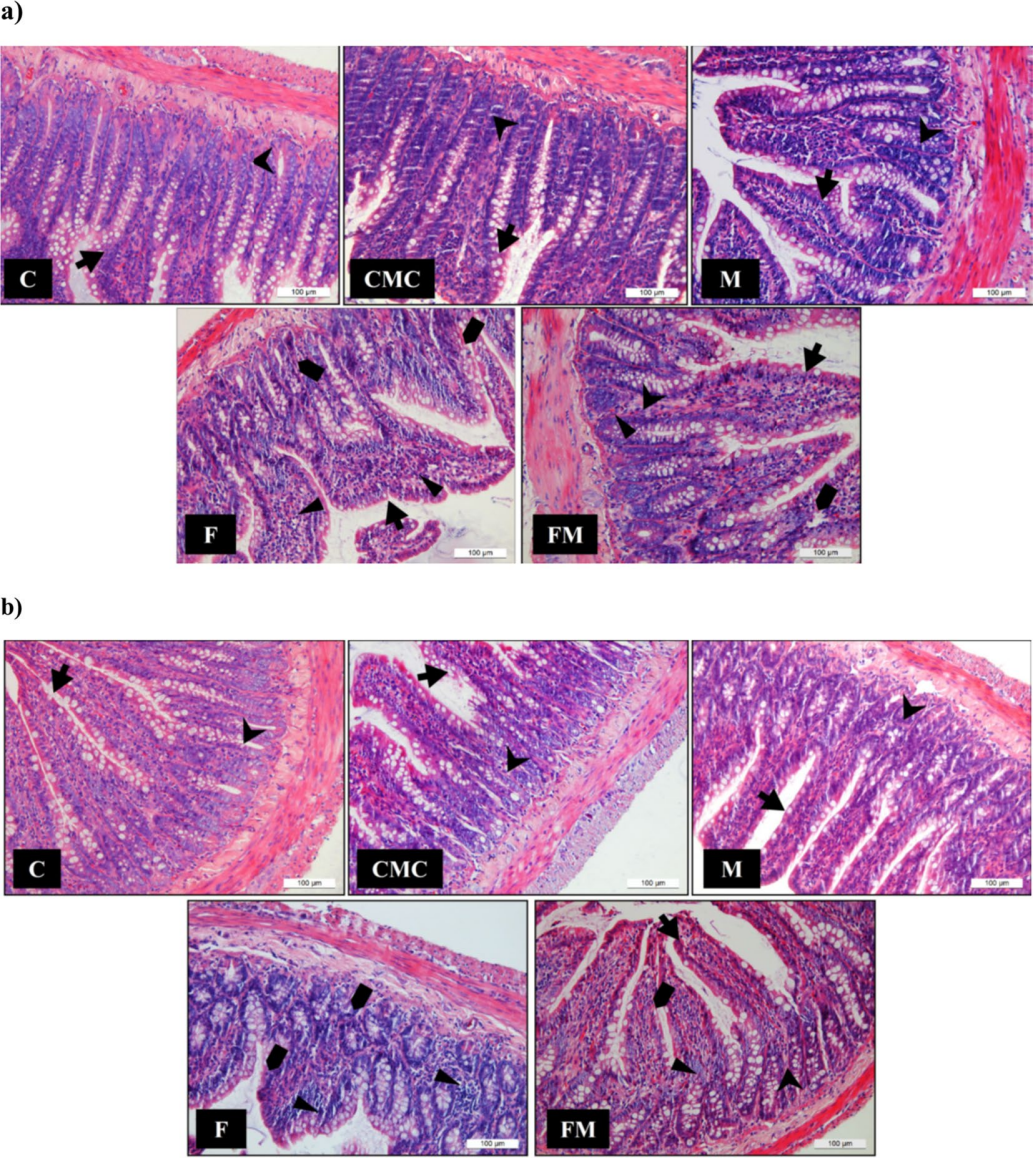
Histopathological observation of the ileum tissue of male (**a**) and female (**b**) rat groups. Epithelial tissue (⬅) and crypt (  ) were observed in all experimental groups. The F and FM groups showed inflammatory cell infiltration (►)and damage to epithelial tissue and crypt structures (➨). Inflammation was absent in the C, CMC, and M groups. Compared with the F group, the ileum of the FM group showed mild inflammatory cells and degeneration of epithelial tissue and crypt structures (Hematoxylin&Eosin, × 200)

As a result of histological scoring of ileum tissues, inflammation and epithelial/crypt damage scores of male and female rats in the F group showed a statistically significant increase compared to the C and FM groups, while no tissue regeneration was observed. It was also noted that in addition to inflammation, degeneration of epithelial tissue and crypt structures decreased (with scoring) in the FM group compared to the F group (Fig. 2a and b).

Figures 2a and 2b show, respectively, that the male and female groups in the ileum tissues. The ileum histological structure was normal, with integrity of the epithelium, villi, and crypts in the C, CMC, and M groups. No pathological changes, as well as the absence of inflammatory infiltrate are in these groups. Fructose-induced epithelial tissue and crypt structures damage and inflammatory cell infiltrate compared to the control groups.

As a result of histological scoring of ileum tissues, inflammation and epithelial/crypt damage scores of male and female rats in the F group showed a statistically significant increase compared to the C and FM groups, while no tissue regeneration was observed. It was also noted that in addition to inflammation, degeneration of epithelial tissue and crypt structures decreased (with scoring) in the FM group compared to the F group (Fig. 2a and b).

### Immunohistochemical analysis findings in ileum tissue of male and female rats

Zonulin, Occludin, Claudin-1, Claudin-2, ZO-1, E-Cadherin, and F-Actin immunoreactivity in ileum tissues of the experimental groups were evaluated by measuring the percentage of immunopositive areas with immunohistochemical analysis.

**Fig. 3:**
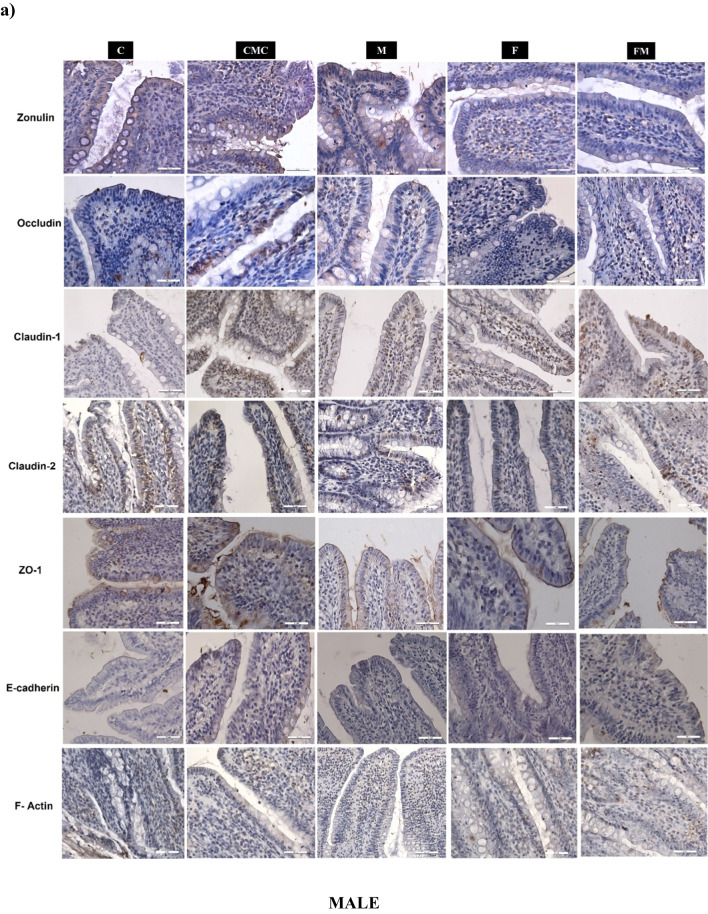

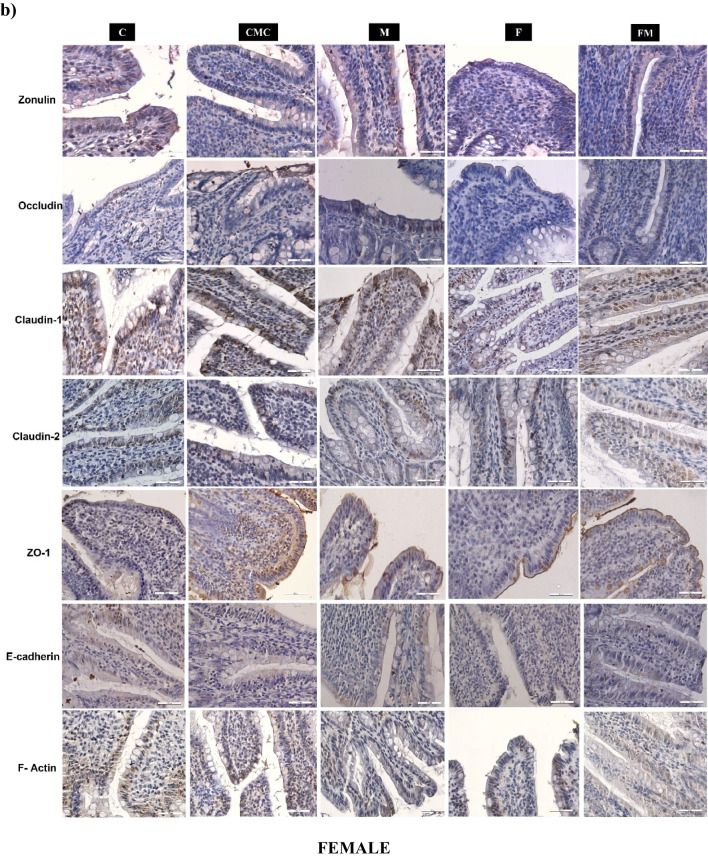
Zonulin, Occludin, Claudin-1, Claudin-2, ZO-1, E-Cadherin and F-Actin immunoreactivity of male (**a**) and female (**b**) rat groups (DAB-Hematoxylin, X400)

There was no statistically significant difference between male and female rat groups in the percentage of Zonulin immunopositive area (Table 5; Fig. 3a and b). The percentage of Zonulin immunopositive area was higher in the F and FM groups in male rats than in female rats (Table 5).

**Table 5:** Zonulin, Occludin, Claudin-1, Claudin-2, ZO-1, E-Cadherin, and F-Actin immunoreactivities in ileum samples of male and female rats (C, *n* = 6; CMC, *n* = 4; M, *n* = 6; F, *n* = 8; FM, *n* = 8). Intergroup analysis: ^*^Different from C group, ^+^different from CMC group, ^#^different from M group, ^&^different from FM group, one-way ANOVA, post-hoc Bonferroni test (*p* < 0.05). Cross-sex analysis: ^a^different from the same group of females, Student t-test (*p* < 0.05)

	C	CMC	M	F	FM
Male					
Zonulin	4.93 ± 0.3	4.48 ± 0.2	4.49 ± 0.2	4.54 ± 0.2^a^	4.61 ± 0.2^a^
Occludin	1.78 ± 0.2^a^	2.11 ± 0.2^a^	2.94 ± 0.3^a^	1.51 ± 0.2^#a^	1.72 ± 0.2^#^
Claudin-1	1.17 ± 0.2^a^	1.90 ± 0.3^a^	1.93 ± 0.2	1.62 ± 0.2	1.66 ± 0.2^a^
Claudin-2	1.94 ± 0.3	1.49 ± 0.2	1.29 ± 0.3	0.55 ± 0.1^*+#&a^	1.69 ± 0.1
ZO-1	1.67 ± 0.2	1.83 ± 0.2	2.63 ± 0.2^*+&^	1.95 ± 0.2^a^	1.65 ± 0.2
E-Cadherin	1.51 ± 0.1	1.63 ± 0.2	1.66 ± 0.2	1.25 ± 0.1^a^	1.54 ± 0.4
F-Actin	2.14 ± 0.2^a^	2.26 ± 0.2^a^	1.91 ± 0.2^a^	1.09 ± 0.2^*+#^	1.64 ± 0.1^a^
Female					
Zonulin	3.95 ± 0.3	4.53 ± 0.3	4.81 ± 0.2	2.95 ± 0.3^+#^	3.86 ± 0.3
Occludin	1.22 ± 0.1	0.76 ± 0.1	0.97 ± 0.2	0.45 ± 0.1^*#&^	1.24 ± 0.1
Claudin-1	3.46 ± 0.4	3.68 ± 0.3	2.10 ± 0.2^*+^	1.81 ± 0.3^*+&^	3.06 ± 0.3
Claudin-2	2.15 ± 0.2	1.66 ± 0.2	1.99 ± 0.2	1.62 ± 0.2	1.97 ± 0.2
ZO-1	2.27 ± 0.2	2.26 ± 0.2	2.09 ± 0.0	1.26 ± 0.1^*+#^	1.74 ± 0.1
E-Cadherin	1.74 ± 0.1	1.79 ± 0.2	1.75 ± 0.2	2.66 ± 0.6	2.01 ± 0.4
F-Actin	1.24 ± 0.2	1.13 ± 0.1	1.22 ± 0.1	1.12 ± 0.2	0.8 ± 0.2

There was no statistically significant difference in the percentage of Occludin and Claudin-1 immunopositive areas between male rat groups (Table 5; Fig. 3a). In female rats, the percentage of Occludin and Claudin-1 immunopositive areas was found to be lower in the F group than the C group and they were higher in the FM group compared to the F group (Table 5; Fig. 3b). When compared between sexes, the percentage of Occludin immunopositive area was found to be higher in males than females in all groups, although it was not significant in the FM group. It was observed that the percentage of Claudin-1 immunopositive area was lower in male C and FM groups than in the same female groups (Table 5).

The percentage of Claudin-2 immunopositive area was lower in the F group of male rats compared to the C and FM groups (Table 5; Fig. 3a), and there was no statistically significant difference between the female rat groups (Table 5; Fig. 3b). It was found that the percentage of Claudin-2 immunopositive area of the male F group was lower than the female F group (Table 5).

There was no statistically significant difference between the groups in the percentage of ZO-1 immunopositive areas in male rats (Table 5; Fig. 3a). In female rats, the percentage of ZO-1 immunopositive areas was lower in the F group compared to the C group (Table 5; Fig. 3b). It was observed that the percentage of ZO-1 immunopositive area was higher in the male F group compared to the female F group (Table 5).

There was no statistically significant difference between the groups in the percentage of E-Cadherin immunopositive area in male and female rats (Table 5; Fig. 3a and b). It was observed that the percentage of E-Cadherin immunopositive area in the male F group was lower than in the same female group (Table 5).

In male rats, the percentage of F-Actin immunopositive area was lower in the F group than in the C group (Table 5; Fig. 3a). In female rats, there was no statistically significant difference between the groups (Table 5; Fig. 3b). The percentage of F-Actin immunopositive area was higher in male rats compared to female rats in the C and FM groups (Table 5).

### Gut microbiota analysis findings

After examining 64 samples (32 in the male group and 32 in the female group), a total of 8 481 079 readings were carried out in these samples. Among male rat groups, while 684 325 readings were obtained in the C group (*n* = 6), 608 752 readings in the CMC group (*n* = 4), 486 629 readings in the M group (*n* = 6), 1 251 101 readings in the F group (*n* = 8) and 807 519 readings in the FM group (*n* = 8), among the female rat groups; 777 755 readings were obtained in the C group (n = 6), 535 473 readings in the CMC group (*n* = 4), 1 692 113 readings in the M group (*n* = 6), 821 912 readings in the F group (*n* = 8) and 815 500 readings in the FM group (*n* = 8).

#### Alpha and beta diversity analysis

In this study, the alpha diversity of gut microbiota obtained from male and female rat groups was interpreted using Chao1 and Shannon indices. These indices are useful tools for understanding species diversity in ecosystems. The Chao1 index provides an estimated species richness by consideringthe impact of rare species, while the Shannon index measures diversity by consideringthe evenness between species.

**Fig. 4:**
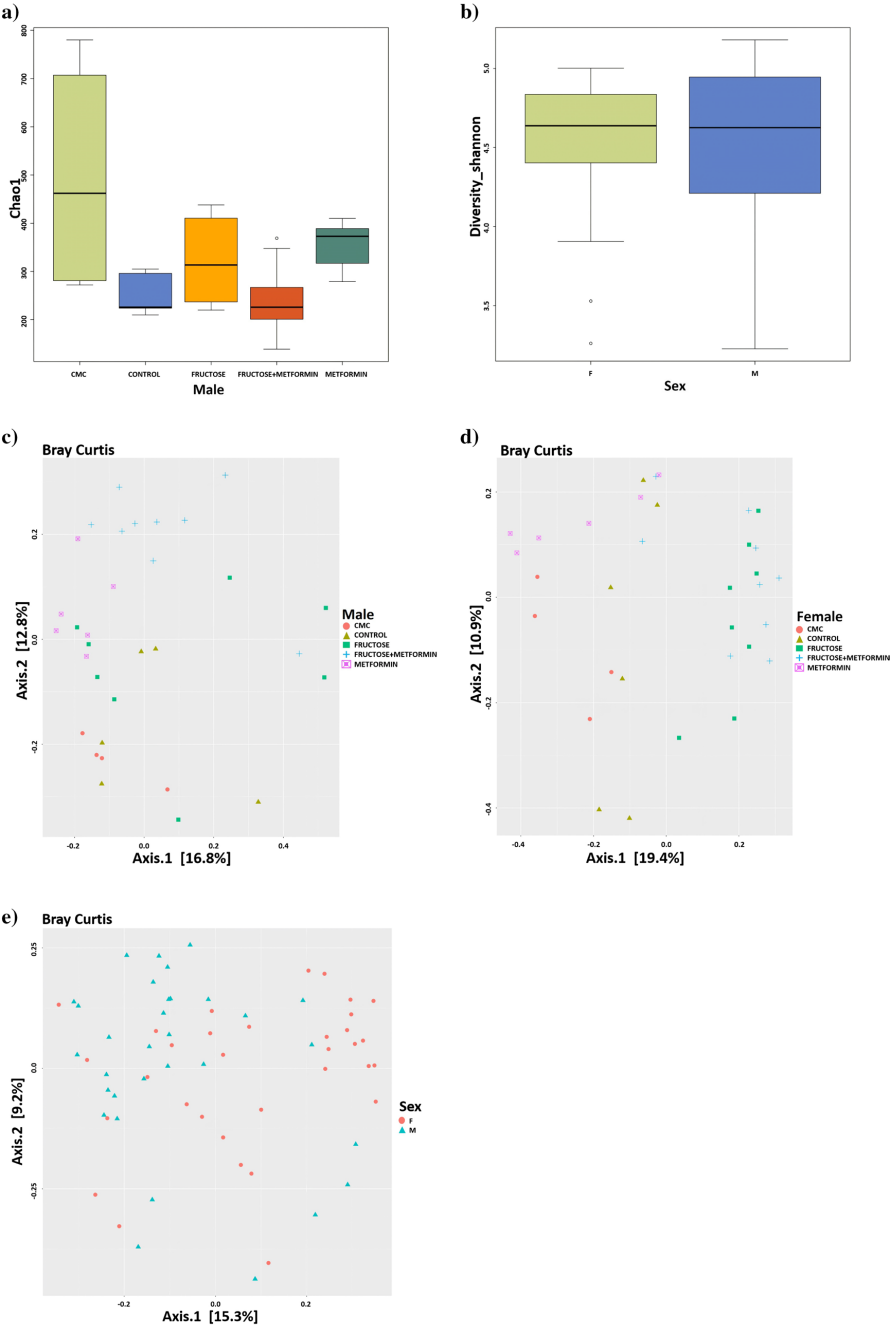
Demonstration of microbiome differences between male and female groups with boxplots and dot plots. Alpha and beta diversity plots to show the differences in microbiota structure between groups (C, *n* = 6; CMC, *n* = 4; M, *n* = 6; F, *n* = 8; FM, *n* = 8); **a** Chao1 diversity indices of the gut microbiota of male rat groups. **b** Shannon diversity indices of the gut microbiota of male/female rat groups (F = Female (Green), M = Male (Blue)) (Boxes indicate first and third quartiles, dashed lines indicate upper and lower whiskers and horizontal thick lines indicate median). **c**-**d**-**e** 2D PCoA plots between groups of male, female, and male/female rats (each dot represents a sample)

According to the Chao1 and Shannon indices, alpha diversity, which refers to ASV richness and microbial abundance, increased in the M group compared to the FM and C groups in males. Although there were changes in other groups in terms of microbial findings, no significant difference was observed (Fig. 4a and Fig. S12a; Tables S2 and S5). In female rat groups, no statistically significant difference was observed (Fig. S11b and S12b; Tables S3 and S6). When the male and female rat groups were compared, it was observed that the diversity scale increased in males and narrowed in females, but it was not statistically significant (Fig. 4b and Fig. S12c; Tables S4 and S7).

Beta diversity analysis used to assess individual taxonomic differences is usually performed using beta diversity indices such as Jaccard, Bray–Curtis, or Sørensen. These indices measure species composition similarities or differences. This study, beta diversity measurement was performed using Principal Coordinates Analysis (PCoA) based on Bray–Curtis distances. According to PCoA, the F group showed different clustering compared to the C group in both male and female rats (Fig. 4c and d). It was observed that the FM group clustered differently compared to the F group in male rats. Metformin altered the gut microbiota of the FM group, causing it to move away from the C group (Fig. 4c). In female rats, there was a close clustering of the F and FM groups and metformin slightly changed the gut microbiota of the FM group, but this was insufficient to approximate it to the C group (Fig. 4d). There was a difference between male and female rats in terms of beta diversity measured by PCoA based on Bray–Curtis distance (Fig. 4e).

Adonis (Table S8), Multiple Response Permutation Procedure (MRPP) (Table S9), Non-Metric Multidimensional Scaling (NMDS) (Table S10), and Anosim (Fig. S13) analyses were used for the statistical analysis of beta diversity sample groupings. According to the analysis results, the groupings differ and are correct to some extent.

Adonis analysis gives information about whether grouping is sufficient or not. Likewise, MRPP analysis is also used for this purpose. When the analyses in Table 8 is examined statistically, it is seen that the p values are significant and the groupings made in our study are sufficient. In MRPP analysis, A values provide information about whether groups have different characteristics; positive values of A indicate that the groups have different properties. According to MRPP analysis, A values are positive, and the groups created in our study have different characteristics (Table S9).

The Anosym analysis confirms the Adonis and MRPP analysis results while also providing information about whether the grouping is valuable. According to Anosym analysis, to say that there is a difference between groups, "R", which can take values between -1 and 1, must have a value above zero. When the R-value takes a value between -1 and 0, the groups are considered to be similar to each other, and when it takes a value between 0 and 1, they are considered to be different from each other; the closer the R-value is to 0, the more similar the groups are to each other. The R values of male rat groups (Fig. S13a), female rat groups (Fig. S13b), and sex-based groups (Fig. S13c), were calculated as 0.24, 0.382, and 0.046, respectively, and it is seen that the groupings are different from each other in our study.

According to NMDS, the grouping is considered correct when the R2 value is above 0.8. According to the NMDS stress analysis based on Bray–Curtis distances, R2 values are above 0.8, and the statistical groupings made in our study were made correctly (Table S10).

When the analysis results are taken together, it is possible to say that the groups created for the study have different characteristics from each other and that the groupings are made correctly and are sufficient.

#### Taxonomic evaluation

**Fig. 5:**
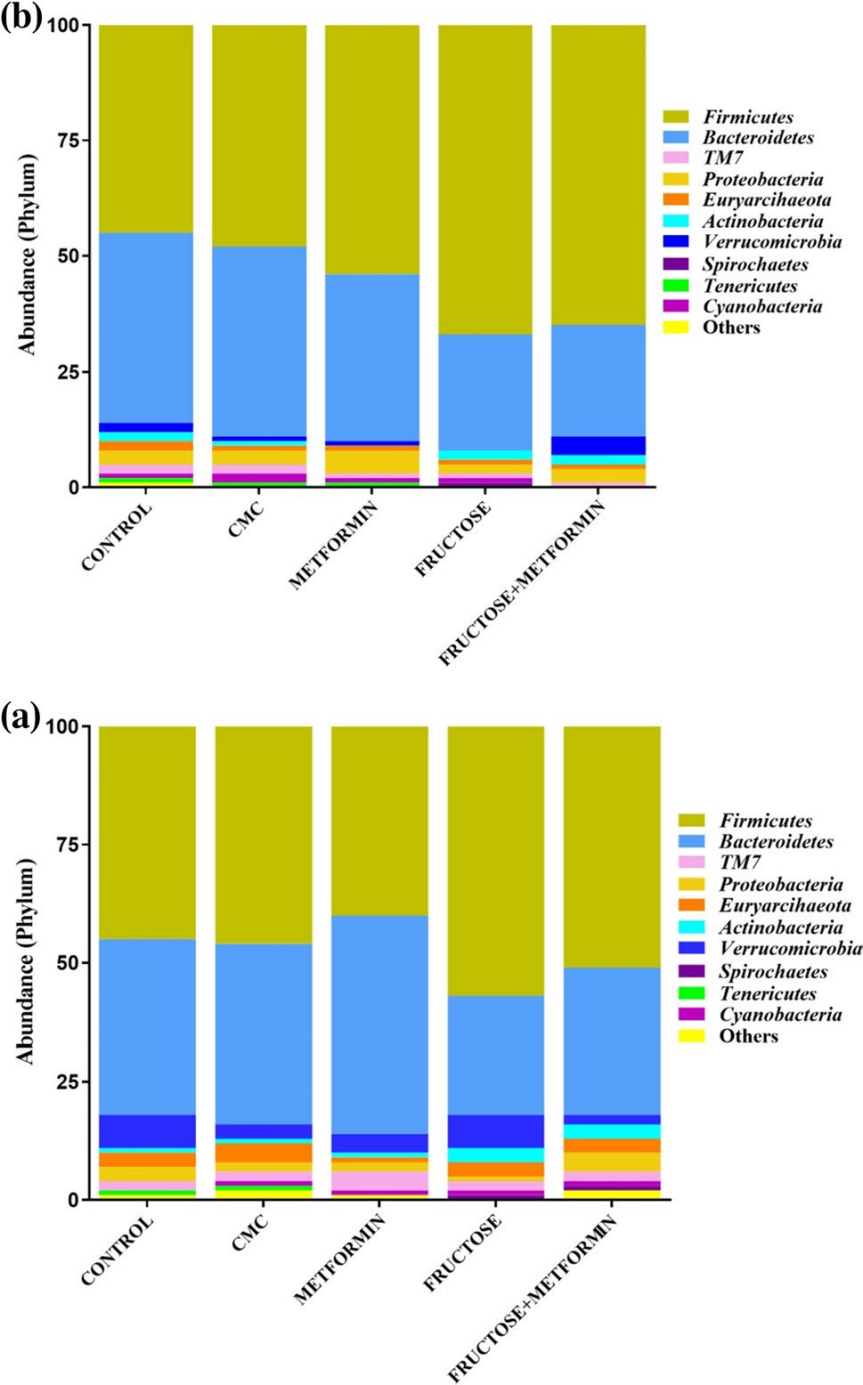
Taxonomic relative abundance distribution at phylum level between a) male and b) female rat groups (C, n = 6; CMC, n = 4; M, n = 6; F, n = 8; FM, n = 8). *TM7*: *Candidatus Saccharibacteria* (*TM7*) is a division under a large bacterial phylum. *Saccharibacteria*, formerly known as *TM7*, is a major bacterial lineage. “Other” is formed by summing the relative abundances of phyla with relative abundances below 1%

Taxonomic bar graphs were used to analyze the composition of the gut microbiota at the phylum level. The taxonomic distribution (abundance) at the phylum level is given in Fig. 5a in male rat groups and in Fig. 5b in female rat groups, respectively.

**Fig. 6:**
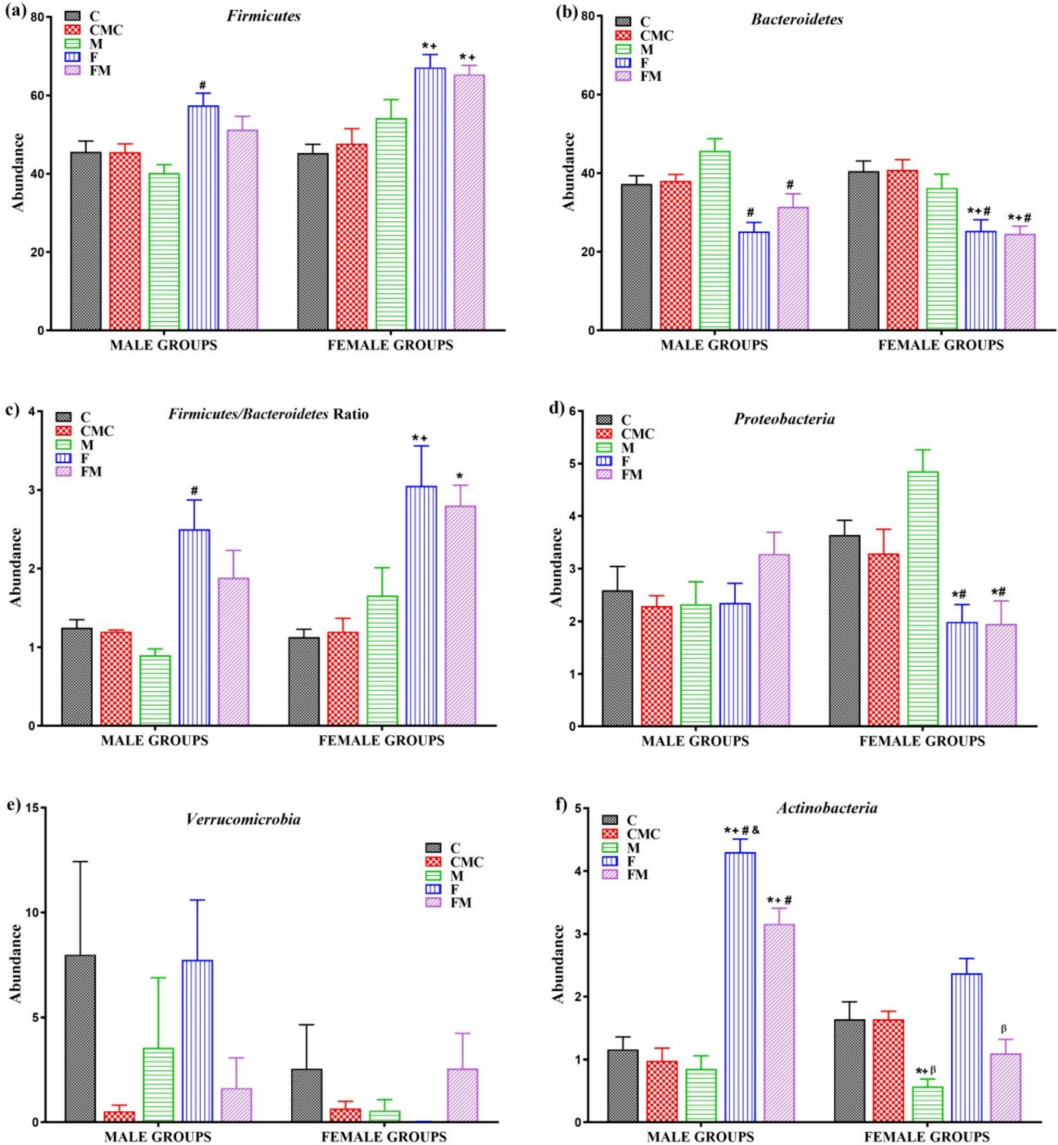
The graphs of **a**) *Firmicutes* **b**) *Bacteroidetes* **c**) F/B ratio **d**) *Proteobacteria* **e**) *Verrucomicrobia,* and **f**) *Actinobacteria* relative abundance in the intestinal microbiota of male and female rat groups (C, n = 6; CMC, n = 4; M, n = 6; F, n = 8; FM, n = 8). Intergroup analysis: ^*^Different from C group, ^+^different from CMC group, ^#^different from M group, ^β^different from F group, ^&^different from FM group, one-way ANOVA, post-hoc Bonferroni test (p < 0.05)

It was observed that the relative abundance of *Firmicutes* phylum was higher in male rats in the F group than in the M group, and in female rats in the F and FM groups compared to the C and CMC groups (Fig. 6a). The relative abundance of the *Bacteroidetes* phylum in the F and FM groups was lower in male rats than in the M group, and in female rats compared to the C, CMC and M groups (Fig. 6b). The F/B ratio of male rats was higher in the F group than in the M group. In female rats, it was found to be higher in the F and FM groups than in the C group and also in the F group compared to the CMC group (Fig. 6c). When compared between sexes, the relative abundance of the *Firmicutes* phylum was lower in male M and FM groups than in the same female groups. There was no significant difference between male and female rat groups in terms of *Bacteroidetes* and F/B ratios (Table S1).

There was no significant difference in the relative abundance of the *Proteobacteria* phylum between male rat groups. The relative abundance of the *Proteobacteria* phylum was found to be higher in the C and M groups of female rats than in the F and FM groups (Fig. 6d). While the relative abundance of the *Proteobacteria* phylum was lower in males compared to females in the M group, it was higher in the FM group (Table S1).

There was no significant difference in the relative abundance of the *Verrucomicrobia* phylum between the groups in male and female rats (Fig. 6e). In females, *Verrucomicrobia* phylum could not be detected in group F as a result of the applied method. Therefore, when compared between sexes, the Verrucomicrobia phylum's relative abundance was higher in the male F group than in the female F group (Table S1).

The relative abundance of the *Actinobacteria* phylum was higher in male rats in the F and FM groups than in the C, CMC, and M groups. In female rats, it was lower in the M group compared to the C, CMC, and F groups. In both male and female rats, it was found to be lower in the FM group than in the F group (Fig. 6f). The relative abundance of the *Actinobacteria* phylum was lower in the CMC group and higher in the F and FM groups in males compared to females (Table S1).

**Fig. 7:**
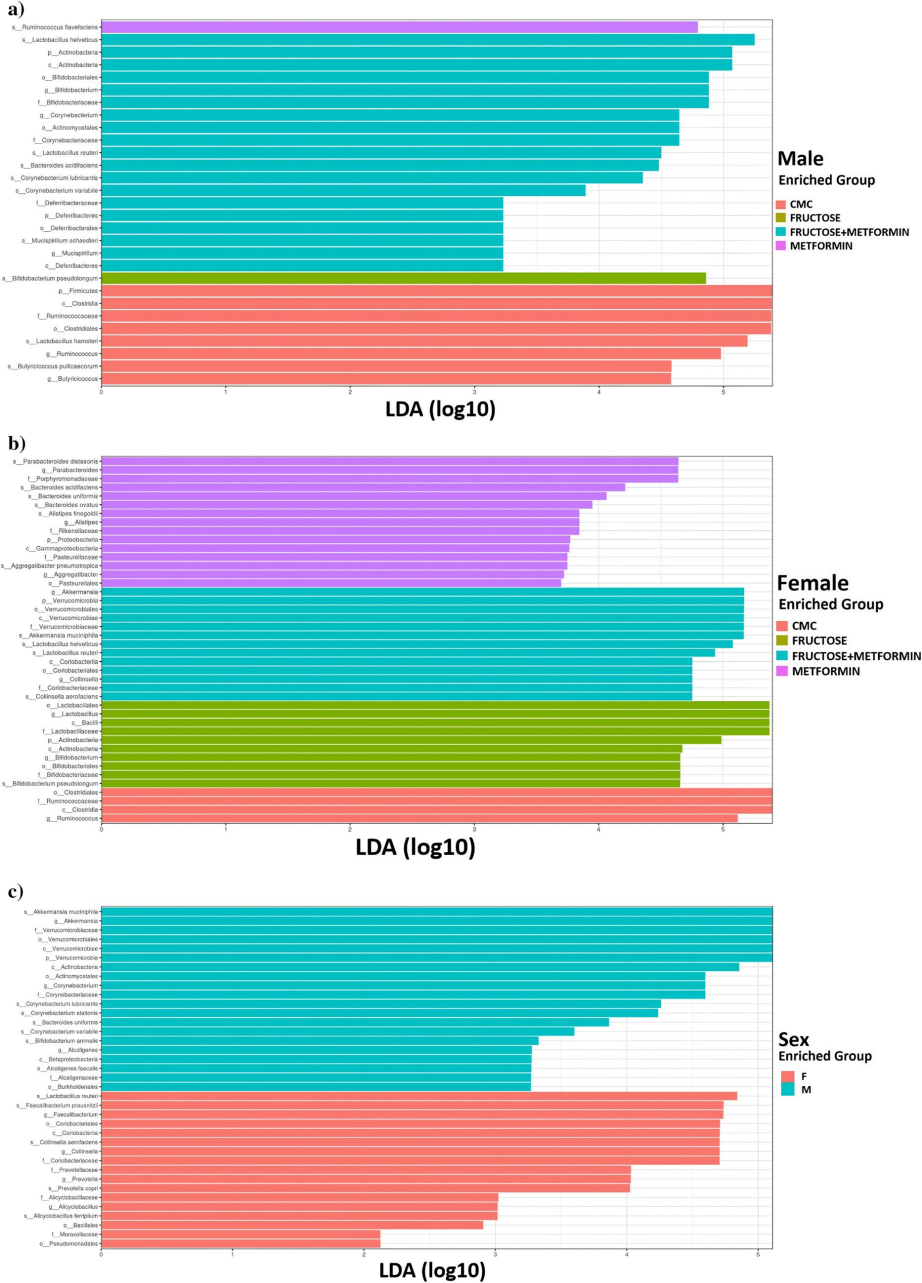
LDA scoring graph created to show significant microorganism differences between male and female rat groups (C, *n* = 6; CMC, *n* = 4; M, *n* = 6; F, *n* = 8; FM, *n* = 8) (LDA score > 2, *P* < 0.05); **a** Male rat groups **b**) Female rat groups **c**) Male and female together

The LEfSe analysis method was applied for the graphical representation of the taxa that were differentially abundant between the groups and their effect sizes and phylogenetic relationships. According to the LEfSe analysis, the graphs created show microbial communities whose relative abundances differ notably at all taxonomic levels in the gut microbiota of male, female, and sex-based rat groups (Fig. 7a, b, c). Among the microbial communities in question, those whose LDA score is higher than the logarithmic threshold value of 2 are defined as biomarkers. In our study, according to LEfSe analysis, at the genus and species level.

In male rats, *Ruminococcus, Butyricococcus, Lactobacillus hamsteri,* and *Butyricococcus pulliaceorium* differed in the CMC group; *Ruminococcus flavefaciens* differed in the M group*; Bifidobacterium pseudolongum* differed in the F group; *Bifidobacterium, Corynebacterium, Mucispirillum, Lactobacillus helveticus, Lactobacillus reuteri, Corynebacterium lubricantis, Corynebacterium variable, Bacteroides acidifaciens,* and *Mucispirillum schaedleri* differed in the FM group (Fig. 7a).

In female rats, *Ruminococcus* differed in the CMC group; *Parabacteroides, Alistipes, Aggregatibacter, Bacteroides acidifaciens, Bacteroides ovatus, Alistipes finegoldi,* and *Aggregatibacter pneumotropica* differed in the M group; *Akkermansia, Collinsella, Akkermansia muciniphilla, Lactobacillus helveticus,* and *Lactobacillus reuteri* differed in the F group (Fig. 7b).

According to the grouping of male and female rats among themselves, *Faecalibacterium, Collinsella, Prevotellave, Alicyclobacillus, Lactobacillus reuteri, Faecalibacterium prausnitzii, Collinsella aerofaciens, Prevotella copri,* and *Alicyclobacillus ferripilum* differed in females; *Akkermansia, Corynebacterium, Alcaligenes, Akkermansia muciniphila, Corynebacterium lubricantis, Corynebacterium stationis, Bacteroides uniformis, Corynebacterium variabile, Bifidobacterium animalis,* and *Alcaligenes faecalis* differed in males (Fig. 7c).

## Discussion

Today, corn syrup containing high levels of fructose is used as a sweetener in ready-to-eat foods. In recent years, the prevalence of MetS has been increasing in parallel with the increase in dietary HF intake [31]. Metformin, an antihyperglycemic agent, is recommended for the first-line pharmacological treatment of MetS [7]. In a study, it has been revealed that oral intake of metformin was more effective in lowering glucose levels than intravenous infusion [32]. It has been suggested that this may be because it reduces glucose levels due to increased metformin concentration in the small intestine [12]. Therefore, it is thought that the gut microbiota, a new target in the mechanisms of action of metformin, may play a role in the therapeutic effect of the drug [10]. However, studies on the effects of sex differences on metformin-mediated gut microbiota changes are limited and insufficient information exists. Our study aimed to reveal possible changes in the gut microbiota with oral administration of metformin in male and female rats created as a MetS model with an HF diet, according to sex difference. Also, the contribution of the gut microbiota to the therapeutic effect of metformin was investigated in the presence of intestinal TJ proteins and inflammation parameters.

Many studies are revealing the effects of HF diet and metformin treatment on the body weight of rodents [33–35]. In this study, HF diet and metformin treatment did not cause a significant difference in weight gain in males. In females, the highest weight gain was observed in the F group, and the lowest weight gain was observed in the M group. Feeding HF diet decreased feed consumption in both sexes. The decrease in feed consumption is probably due to the fact that calorie needs are met by fructose. Metformin alone did not affect feed intake in males but decreased it in females. The lower feed intake and decrease in body weight are likely due to the appetite-reducing effect of metformin being more prominent in females. While body weight in male Wistar rats fed an HF diet increased in the study of Yin et al. [35], it did not change significantly in the study of Kaseda et al. [36], similar to our results. Metformin, unlike most glucose-lowering drugs, does not cause weight gain or causes mild weight loss [37]. While metformin treatment reduced body weight gain in High-Fat Diet (HFD)-fed male Wistar rats [17], consistent with our results, it did not show a significant effect on body weight gain in the study conducted on male and female mice [33, 34].

According to our study results, glucose, insulin, and HOMA-IR values increased with HF in both sexes and decreased in response to metformin treatment, as expected. In previous studies, glucose [35], insulin [36, 38], and HOMA-IR [38, 39] values increased with an HF diet in male Wistar rats, while they decreased with metformin treatment in male Wistar rats with type 2 diabetes mellitus (T2DM) [40]. In this study, it is noteworthy that the glucose-lowering effect was more prominent in females. A similar result obtained with another study [33] was explained by the protective effect of female hormones in glucose and lipid metabolism against metabolic disorders.

According to our study results, fructose caused a significant increase in serum TG, which is a determinant for the development of MetS in both sexes. It is a remarkable finding that the TG decreased in both sexes with metformin treatment, but this decrease was more prominent in males. In the study conducted by Dupas et al. [39] on Wistar rats, 21-week fructose supplementation resulted in higher TG levels, unlike their previous studies [38], in which the TG level did not change with 6 and 12 weeks of fructose supplementation. Our findings suggest that the administration of 20% fructose for 15 weeks is sufficient to establish the MetS model in rats fully.

Fructose-induced MetS has been shown to be closely associated with inflammation in the intestine, characterized by increased concentration of pro-inflammatory cytokines [3]. Modulation of the inflammatory signaling pathway by inhibition of pro-inflammatory cytokines is a potential mechanism to reduce inflammation. Metformin is known to directly suppress the release of proinflammatory cytokine such as IL-1β and TNF-α [14]. In the study of Do et al. [41], an HF diet caused an increase in IL-1β and TNF-α in the intestine of male mice. In the study of Ahmadi et al. [42], it was observed that the increased expression of IL-1β and TNF-α in the intestine of HFD-fed male mice decreased with metformin treatment. In this study, it is an essential finding that IL-1β, which increased with HF in both sexes, decreased with metformin treatment in only males, suggesting that females did not benefit from metformin treatment as much as males on this parameter. TNF-α, which did not change between female groups, increased in males fed with HF and decreased in response to metformin treatment. When all these results are considered together, it is possible to say that the effect of the HF diet on the increase in proinflammatory cytokine levels and the response to metformin treatment is more prominent in males.

HF diet is known to increase inflammatory cell infiltration, a sign of inflammation [43]. In this study, due to histological scoring of ileum tissues, inflammation, epithelial, and crypt damage of male and female rats fed HF were higher compared to rats in other groups, and no tissue regeneration was observed. Notably, in addition to inflammation, degeneration in epithelial tissue and crypt structures decreased in response to metformin treatment in both sexes. The inflammation score was lower in females compared to males in the FM group; this finding contrasts with the finding in the previous paragraph, which showed that males respond better to metformin treatment.

The excessive inflammatory response in the small intestine leads to abnormal epithelial morphology [8]. In this study, with metformin administration, the increase in villus length between male and female groups and a significant increase in crypt depth in females support that metformin contributes positively to the morphological changes in the ileum. These changes may affect the digestive enzyme effect and nutrient transport depending on the increase in the absorptive area.

Intestinal inflammation is known to impair epithelial barrier function, and metformin has been shown to have beneficial effects in alleviating inflammation and barrier destruction [8]. The integrity of the gut barrier is maintained mainly by the structure of TJs and adherens junctions of the mucosal layer. Lipopolysaccharides (LPS) are proinflammatory mediators from different types of gram-negative bacteria and are essential to their outer cell walls. They can change the integrity of the intestinal barrier by dysregulating the TJ proteins, leading to a gut barrier injury through an inflammatory response. The impaired intestinal barrier integrity permits bacteria-derived molecules and other antigens to cross the gut barrier to maintain this intestinal inflammation. LPS can enter the circulatory system and trigger inflammatory-immune responses after bacterial release in pathological conditions [44].

In this study, ZO-1 in females and F-Actin in males decreased with HF. Also, Occludin and Claudin-1, which decreased with HF in females and Claudin-2, which decreased with HF in males, increased with metformin treatment. These proteins were observed to change more significantly in females with HF diet and metformin treatment, which may indicate that females are more susceptible to HF diet in gut permeability and respond better to metformin treatment. In previous studies, feeding an HF diet reduced the expression of Occludin, Claudin-1, and ZO-1 in the intestine of female rats [45] and Claudin-2 in the ileum of female mice [46]. Similar to our study results, it was observed that the expression of Occludin, Claudin-1, and ZO-1, which decreased with HFD and streptozotocin injection in the ileum of male rats, increased with metformin treatment [47, 48].

In rats fed a fructose-rich diet, it is suggested that the development of MetS is directly related to variations in the gut content of specific bacterial taxa [49]. The diversity and composition of the gut microbiota significantly changed during metformin treatment [33]. Recently, the vaginal microbiota has been shown to vary significantly by menopausal status, suggesting that hormones in females are highly correlated with the composition of the human vaginal microbiota. Therefore, it can be suggested that human hormone levels may similarly affect the human gut microbiota. Thus, differences in the gut microbiota between males and females during metformin treatment may result from differences in hormone levels [33].

Microbiota diversity is thought to indicate a "healthy gut" [47]. In this study, in both sexes the alpha diversity of the gut microbiota did not change depending on HF diet or metformin treatment indicating that the number of species also did not change. In a study evaluating the effect of an HF diet on the gut microbiota, a loss of intestinal microbial diversity was observed in male mice fed an HF diet [41]. Alpha diversity in HFD-fed male mice, where the effect of metformin treatment on the gut microbiota was investigated, was analyzed using Chao 1 and Shannon indices, and no significant difference was found between the groups, similar to our study results [16]. According to our beta diversity results, there is a clear clustering depending on HF diet and metformin treatment in both sexes. Especially in females, although metformin slightly changed the gut microbiota composition of the FM group, it was insufficient in terms of approximation to the C group; that is, metformin did not alter the gut microbiota at a level that would eliminate the disease. In Do et al.'s [41] study, PCoA revealed that fructose-fed mice had a different microbial composition that caused them to cluster separately from normal diet-fed mice. Also, PCoA results in female and male mice, in which HFD induced metabolic disorders, showed that the HFD-fed and metformin-treated groups were clearly separated from each other [33]. In the study of Silamiķele et al. [34], while there was a clear clustering based on PCoA, diet, and sex, no clear clustering was observed regarding metformin treatment status.

The microbiota components are mostly bacteria with a small number of viruses, fungi, and eukaryotic cells. In both humans and mice, the most abundant phyla are *Firmicutes*, which make up 60–80%, *Bacteroidetes*, which make up 20–30%, and *Actinobacteria,* representing a minority of approximately 10%, while the *Proteobacteria* phylum is present in lesser proportions (< 1%) [50]. *Firmicutes* and *Bacteroidetes* represent more than 90% of the relative abundance of the gut microbiome and their interactions play an important role in maintaining gut homeostasis [51]. In this study, *Firmicutes* and *Bacteroidetes* phyla were dominant in both male and female groups, as expected. The HF diet caused an increase in the *Firmicutes* and a decrease in the *Bacteroidetes* in females. The F/B ratio, an indicator of gut permeability, is associated with the maintenance of gut homeostasis, and changes in this ratio can lead to various pathologies [52]. For example, an increase in the F/B ratio in the cecum microbiota was shown to be associated with obesity and T2DM [53]. It was observed that the F/B ratio increased in male mice fed the HF diet compared to mice fed the normal diet [41]. The decrease in the F/B ratio is accepted as a predictor of metabolic improvement with metformin treatment [16]. Metformin treatment caused a decrease in the F/B ratio in male mice fed an HFD [16, 54]. In this study, it is an important finding that the increase in the F/B ratio with HF diet was observed only in females. This result indicates that the HF diet may impair permeability and homeostasis of the gut in females.

*Actinobacteria* and *Proteobacteria* represent 10% of the relative abundance of the gut microbiome. *Actinobacteria* plays a vital role in maintaining gut homeostasis [51], and the *Proteobacteria* plays an essential role in inflammation [55]. In this study, the HF diet caused a decrease in the *Proteobacteria* in females. The *Actinobacteria* increased with HF diet while decreasing with metformin treatment in males. *Verrucomicrobia* did not change with HF diet and metformin treatment in both sexes. An HF diet caused a decrease in the relative abundance of the phyla *Proteobacteria*, *Actinobacteria,* and *Verrucomicrobia* in male Wistar rats [56]. In previous studies, the relative abundance of the phyla *Verrucomicrobia* [33] and *Actinobacteria* [34] in HFD-fed male and female mice and *Proteobacteria* in HFD-fed male Wistar rats [17] increased in response to metformin treatment. However, the phylum is the higher taxonomic order consisting of some pathogens as well as some beneficial bacteria, so it is recommended to investigate lower taxonomic orders, such as the abundance of genus and species levels, to understand the role of the microbiome on metabolic outcomes [53]. In this study, it was possible to determine the microbial communities defined as biomarkers in the gut microbiota of rat groups, the relative abundance of which differed significantly at all taxonomic levels with LEfSe Analysis. It is noteworthy that in the disease group, *Bifidobacterium pseudolongum* and the treatment group, *Lactobacillus helveticus,* and *Lactobacillus reuteri* were the prominent common species in both sexes. In previous studies, *Bifidobacterium pseudolongum* was found to be abundant in non-metformin-treated obese rats according to LEfSe analysis [57]*. Lactobacillus reuteri* was significantly higher in fecal samples of diabetic patients than in those of control subjects [58], unlike our study result. *Akkermansia muciniphila*, whose relative abundance increases with metformin treatment, has an anti-inflammatory effect in the intestine. In this context, there may be a negative correlation between the increased *Akkermansia muciniphila* abundance with metformin treatment and inflammatory parameters such as inflammatory cytokines [14]. Therefore, when male and female rat groups were compared in our study, it is possible that *Akkermansia muciniphila*, which was prominent in the male treatment group, was responsible for the improved inflammation parameters observed in males compared to females.

In our study, statistically significant results could not be obtained in microbiota analysis due to the low reading quality; further analysis should enable the identification of specific bacteria that may be associated with changes in the gut microbiota. Correlation analysis should be performed to determine the relationship between modulation of the gut microbiota by metformin and improvement in metabolic parameters.

In our study, ileum tissue was used to evaluate the effect of HF consumption and metformin treatment on inflammation parameters. Chronic low-grade inflammation in the intestine is associated with disruption of intestinal TJs, and low TJ protein expression increases intestinal permeability; thus, LPS produced by Gram (-) bacteria can be absorbed in larger amounts [47]. The level of LPS in blood has previously been linked to systematic inflammation and various types of cancer [44]. Therefore, systemic inflammation should be evaluated by looking at the blood levels of the relevant parameters and LPS level. In addition, the results obtained by ELISA analysis in ileum tissue should be supported by Western Blot analysis.

Our immunohistochemical analysis results, which reveal the changes in TJ proteins between groups, show that HF diet may have an effect on intestinal permeability and barrier function. The fructose-metformin relationship in maintaining intestinal homeostasis and the mucosal barrier should be evaluated together with the gene expressions of cellular connection proteins, and the reasons for the differences in sex groups should be elucidated.

Our study results support that metformin causes morphological changes in the ileum, and these changes may affect the digestive enzyme effect and nutrient transport due to the increase in the absorptive area; in this context, investigating the effects of metformin on GLUT2 and GLUT 5, which are fructose transporters, is essential in terms of elucidating its mechanisms of action.

## Conclusion

In this study, the modulatory role of metformin on the gut microbiota of male and female rats in which dietary HF induced MetS in the presence of intestinal TJ proteins and inflammation parameters was investigated for the first time.

In our study, the fact that metformin alone causes weight loss only in females is essential in revealing the sex difference. According to our biochemical analysis results, it is noteworthy that the decrease in glucose levels with metformin treatment is more pronounced in females, while the decrease in TG levels is more pronounced in men. Also, it is possible to say that the effect of HF diet and metformin treatment on pro-inflammatory cytokine levels is more pronounced in men. Our findings on TJ proteins may indicate that females are more sensitive to the HF diet in terms of intestinal permeability and respond better to metformin treatment. It is an important finding that the increase in the F/B ratio indicates that the HF diet may disrupt intestinal permeability and homeostasis in females; this finding may also explain why females are more sensitive to the HF diet in terms of intestinal permeability. It is noteworthy that *Bifidobacterium pseudolongum* in the disease group and *Lactobacillus helveticus* and *Lactobacillus reuteri* in the treatment group are the most common species in both sexes. Additionally, it is possible that *Akkermansia muciniphila*, which was prominent in the male treatment group, can be responsible for the improved inflammation-related parameters observed in men compared to females.

In conclusion, more comprehensive studies, including both sexes are needed to support that modulation of the gut microbiota with metformin in HF-induced MetS may be one of the mechanisms that may contribute to the therapeutic effect of metformin.

## Supplementary Information

Below is the link to the electronic supplementary material.

Supplementary file1 (DOCX 1465 KB)

## Data Availability

The data used and/or analyzed during the current study are available from the corresponding author on reasonable request.
