# Supplementary material for: Investigation of the relationship between inflammation and microbiota in the intestinal tissue of female and male rats fed with fructose: Modulatory role of metformin
Source: Daru. 2024 Jun 17;32(2):515–35. doi: 10.1007/s40199-024-00521-2 (PMC11554967; doi:10.1007/s40199-024-00521-2)
Supplement: Supplementary file 1 — Supplementary file1 (DOCX 1465 KB) [file 40199_2024_521_MOESM1_ESM.docx]

**Supplementary** **Material**

**Tables:**

| **Groups** | **Sex** | **Firmicutes** | **Bacteroidetes** | **F/B** | **Proteobacteria** | **Verrucomicrobia** | **Actinobacteria** |
| --- | --- | --- | --- | --- | --- | --- | --- |
| **C** | Male | 45.6±2.7 | 37.2±2.1 | 1.3±0.1 | 2.6±0.5 | 8.0±4.4 | 1.2±0.2 |
|  | Female | 45.2±2.3 | 40.5±2.6 | 1.1±0.1 | 3.6±0.3 | 2.6±2.1 | 1.6±0.3 |
| **CMC** | Male | 46.5±2.1 | 38.0±1.6 | 1.2±0.0 | 2.3±0.2 | 0.5±0.3 | 1.0±0.2^a^ |
|  | Female | 47.7±3.8 | 40.8±2.6 | 1.2±0.2 | 3.3±0.5 | 0.7±0.3 | 1.6±0.1 |
| **M** | Male | 40.2±2.1^a^ | 45.7±3.1 | 0.9±0.1 | 2.3±0.4^a^ | 3.6±3.3 | 0.9±0.2 |
|  | Female | 54.2±4.8 | 36.2±3.5 | 1.7±0.4 | 4.9±0.4 | 0.6±0.5 | 0.6±0.1 |
| **F** | Male | 57.4±3.1 | 25.2±2.3 | 2.5±0.4 | 2.4±0.4 | 7.7±2.9^a^ | 4.3±0.2^a^ |
|  | Female | 67.1±3.3 | 25.3±2.8 | 3.1±0.5 | 2.0±0.3 | 0.0±0.0 | 2.4±0.2 |
| **FM** | Male | 51.3±3.4^a^ | 31.4±3.3 | 1.9±0.4 | 3.3±0.4^a^ | 1.6±1.4 | 3.2±0.3^a^ |
|  | Female | 65.4±2.3 | 24.6±1.9 | 2.8±0.3 | 2.0±0.4 | 2.6±1.7 | 1.1±0.2 |

**Table S1**. Comparison of the relative abundances of the phyla Firmicutes, Bacteroidetes, Proteobacteria, Verrucomicrobia, Actinobacteria, and F/B ratios of male and female rat groups according to sex (C, n=6; CMC, n=4; M, n=6; F, n=8; FM, n=8). Cross-sex analysis: ^a^Different from the same group of females, Student t-test (p<0.05).

|  | **CMC** | **FM** | **F** | **C** | Male |
| --- | --- | --- | --- | --- | --- |
| **FM** | 0.112 | - | - | - |  |
| **F** | 0.525 | 0.232 | - | - |  |
| **C** | 0.370 | 0.765 | 0.370 | - |  |
| **M** | 0.914 | 0.048 | 0.765 | 0.087 |  |

**Table S2.** Pairwise comparisons of the Chao1 diversity indices of the gut microbiota of male rat groups (C, n=6; CMC, n=4; M, n=6; F, n=8; FM, n=8) using the "Wilcoxon rank sum exact" test.

|  | **CMC** | **F** | **FM** | **C** | Female |
| --- | --- | --- | --- | --- | --- |
| **F** | 0.94 | - | - | - |  |
| **FM** | 0.94 | 0.94 | - | - |  |
| **C** | 0.94 | 0.94 | 0.94 | - |  |
| **M** | 0.94 | 0.94 | 0.94 | 0.94 |  |

**Table S3.** Pairwise comparisons of the Chao1 diversity indices of the gut microbiota of female rat groups (C, n=6; CMC, n=4; M, n=6; F, n=8; FM, n=8) using the "Wilcoxon rank sum exact" test.

|  | **Female** | Sex |
| --- | --- | --- |
| **Male** | 0.27 |  |

**Table S4.** Pairwise comparisons of the Chao1 diversity indices of the gut microbiota according to sex in rats (C, n=6; CMC, n=4; M, n=6; F, n=8; FM, n=8) using the "Wilcoxon rank sum exact" test.

|  | **CMC** | **FM** | **F** | **C** | Male |
| --- | --- | --- | --- | --- | --- |
| **FM** | 0.189 | - | - | - |  |
| **F** | 0.658 | 0.591 | - | - |  |
| **C** | 0.189 | 0.914 | 0.591 | - |  |
| **M** | 0.914 | 0.088 | 0.914 | 0.088 |  |

**Table S5.** Pairwise comparisons of the Shannon diversity indices of the gut microbiota of male rat groups (C, n=6; CMC, n=4; M, n=6; F, n=8; FM, n=8) using the "Wilcoxon rank sum exact" test.

|  | **CMC** | **F** | **FM** | **C** | Female |
| --- | --- | --- | --- | --- | --- |
| **F** | 0.69 | - | - | - |  |
| **FM** | 0.94 | 0.69 | - | - |  |
| **C** | 0.69 | 0.77 | 0.69 | - |  |
| **M** | 0.69 | 0.69 | 0.77 | 0.65 |  |

**Table S6.** Pairwise comparisons of the Shannon diversity indices of the gut microbiota of female rat groups (C, n=6; CMC, n=4; M, n=6; F, n=8; FM, n=8) using the "Wilcoxon rank sum exact" test.

|  | **Female** | Sex |
| --- | --- | --- |
| **Male** | 0.82 |  |

**Table S7.** Pairwise comparisons of Shannon diversity indices of gut microbiota according to sex in rats (C, n=6; CMC, n=4; M, n=6; F, n=8; FM, n=8) using the "Wilcoxon rank sum exact" test.

| **Group name** | **Df** | **SumOfSqs** | **R2** | **F** | **Pr(>F)** |
| --- | --- | --- | --- | --- | --- |
| **Sex** | 1 | 0.4907 | 0.02661 | 1.722 | 0.023 ‘.’ |
| **Male Groups** | 4 | 1.9087 | 0.22499 | 1.9596 | 0.001‘**’ |
| **Female Groups** | 4 | 2.2437 | 0.25079 | 2.3432 | 0.001‘**’ |
| Significance: 0 ‘***’ 0.001 ‘**’ 0.01 ‘*’ 0.05 ‘.’ 0.1 ‘ ’ 1 | | | | | |

**Table S8.** Adonis analysis of male and female rat groups (C, n=6; CMC, n=4; M, n=6; F, n=8; FM, n=8).

|  | **A** | **Observed delta** | **Expected delta** | **Significance of delta** |
| --- | --- | --- | --- | --- |
| **Sex** | 0.002756 | 1.95E+07 | 1.95E+07 | 0.257 |
| **Male Groups** | 0.03929 | 1.73E+07 | 1.80E+07 | 0.048 |
| **Female Groups** | 0.1591 | 1.74E+07 | 2.07E+07 | 0.003 |

**Table S9.** MRPP analysis of male and female rat groups (C, n=6; CMC, n=4; M, n=6; F, n=8; FM, n=8).

| **Groups** | **R^2^ values lf** |
| --- | --- |
| **Sex** | 0.828 |
| **Male Groups** | 0.912 |
| **Female Groups** | 0.874 |

**Table S10.** NMDS stress analysis of male and female rats (C, n=6; CMC, n=4; M, n=6; F, n=8; FM, n=8).

|  | **Feed Consumption (g/day)** | | **Water Consumption (mL/day)** | |
| --- | --- | --- | --- | --- |
| **Groups** | **Male** | **Female** | **Male** | **Female** |
| C | 25.93±0.7^a^ | 17.39±0.6 | 71.03±1.2^a^ | 42.61±0.7 |
| CMC | 23.79±0.6 ^a^ | 16.65±1.3 | 70.96±2.5 ^a^ | 41.77±0.7 |
| M | 23.23±0.6 ^a^ | 14.98±0.3^*^ | 68.94±0.7 ^a^ | 42.16±1.6 |
| F | 13.29±0.3^*+# a^ | 10.01±0.1^*+#^ | 76.72±2.2^# a^ | 49.04±1.1^*+#^ |
| FM | 11.87±0.7^*+#^ | 10.58±0.3^*+#^ | 78.99±0.7^*+# a^ | 47.89±1.1^*+#^ |

**Table S11.** Daily feed and fluid consumption of male and female rat groups (C, n=6; CMC, n=4; M, n=6; F, n=8; FM, n=8). Intergroup analysis: ^*^Different from the control group, different from the ^+^CMC group, ^#^different from the Metformin group, one-way ANOVA, post-hoc Bonferroni test (p<0.05). Cross-sex analysis: ^a^Different from the same group of females, Student t-test (p<0.05).

**Figures:**


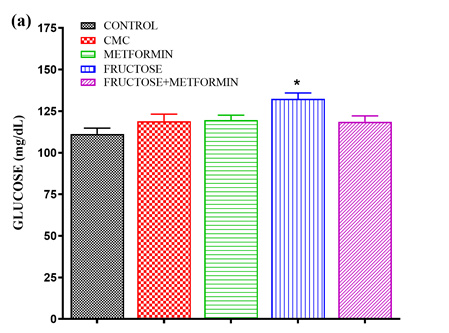

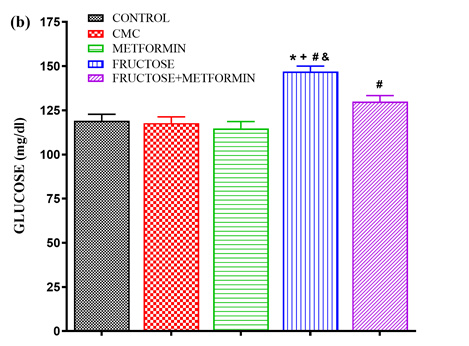


**Fig. S1.** Serum glucose levels (mg/dL) of male (a) and female (b) rats (C, n=6; CMC, n=4; M, n=6; F, n=8; FM, n=8). Intergroup analysis: ^*^Different from C group, ^+^different from CMC group, ^#^different from M group, ^&^different from FM group, one-way ANOVA, post-hoc Bonferroni test (p<0.05).


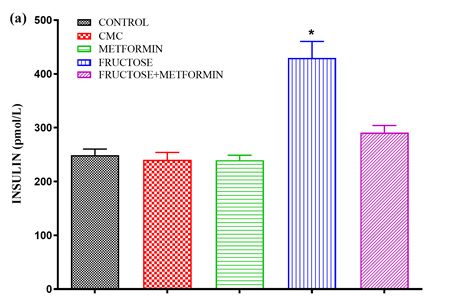

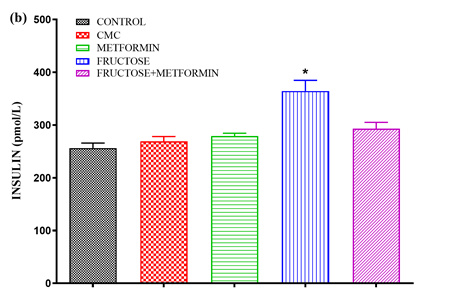


**Fig. S2.** Serum insulin levels (pmol/L) of male (a) and female (b) rats (C, n=6; CMC, n=4; M, n=6; F, n=8; FM, n=8). Intergroup analysis: ^*^Different from all other groups, one-way ANOVA, post-hoc Bonferroni test (p<0.05).


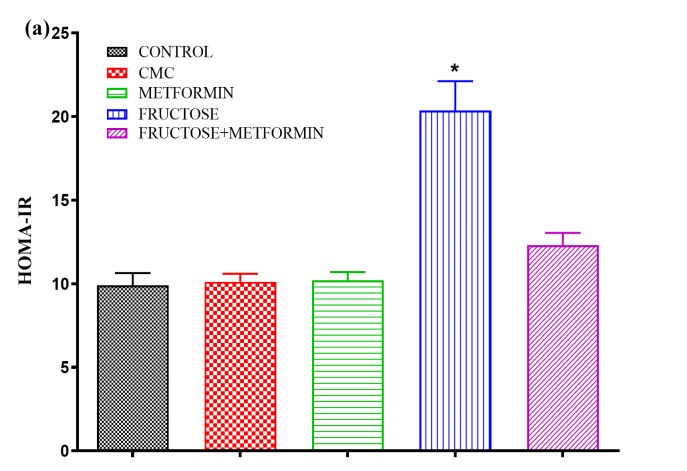

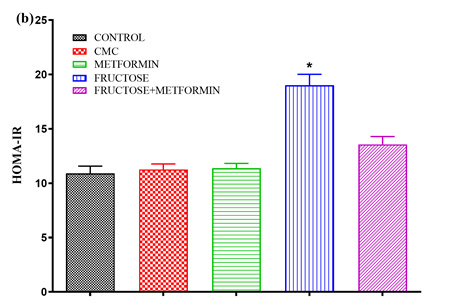


**Fig. S3.** HOMA-IR values of male (a) and female (b) rats (C, n=6; CMC, n=4; M, n=6; F, n=8; FM, n=8). Intergroup analysis: ^*^Different from all other groups, one-way ANOVA, post-hoc Bonferroni test (p<0.05).


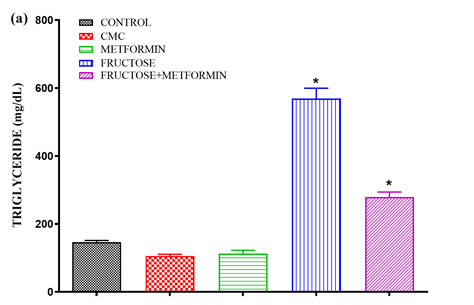

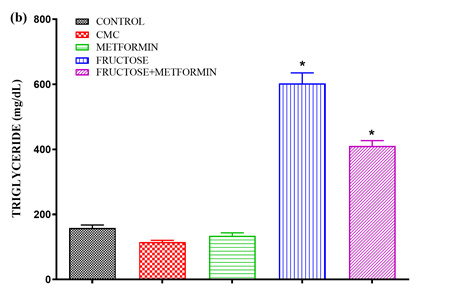


**Fig. S4.** TG values (mg/dL) of male (a) and female (b) rats (C, n=6; CMC, n=4; M, n=6; F, n=8; FM, n=8). Intergroup analysis: ^*^Different from all other groups, one-way ANOVA, post-hoc Bonferroni test (p<0.05).


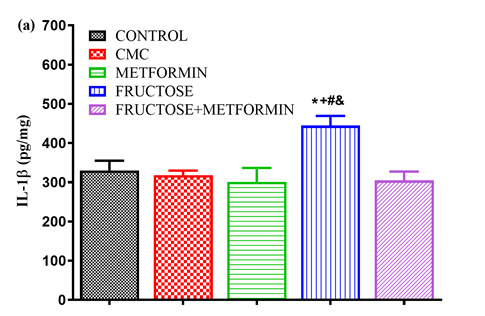

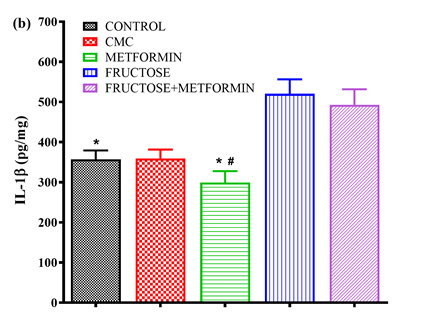


**Fig. S5.** IL-1β levels (pg/mg) measured in ileum tissue of male (a) and female (b) rats (C, n=6; CMC, n=4; M, n=6; F, n=8; FM, n =8). Intergroup analysis: ^*^Different from F group, ^#^different from FM group, one-way ANOVA, post-hoc Bonferroni test (p<0.05).


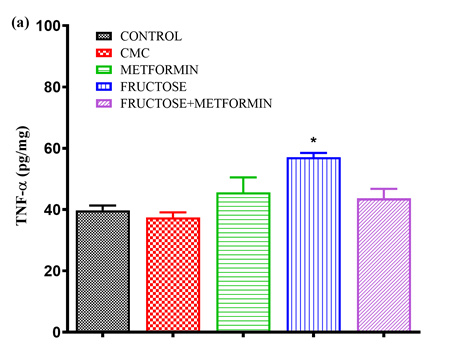

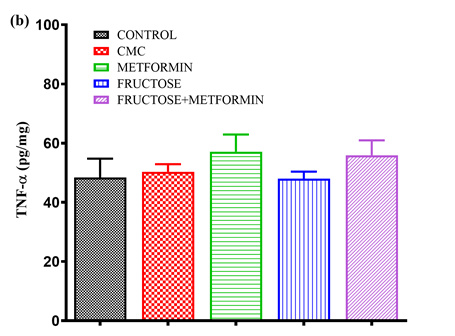


**Fig. S6.** TNF-α levels (pg/mg) measured in ileum tissue of male (a) and female (b) rats (C, n=6; CMC, n=4; M, n=6; F, n=8; FM, n =8). Intergroup analysis: ^*^Different from C, CMC, and FM groups, one-way ANOVA, post-hoc Bonferroni test (p<0.05).


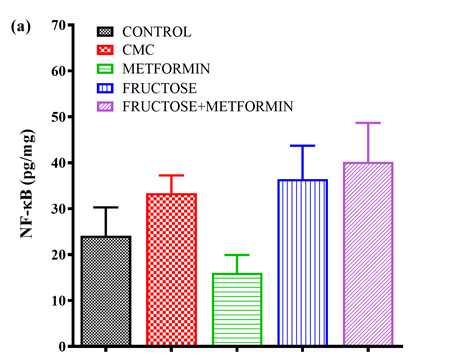

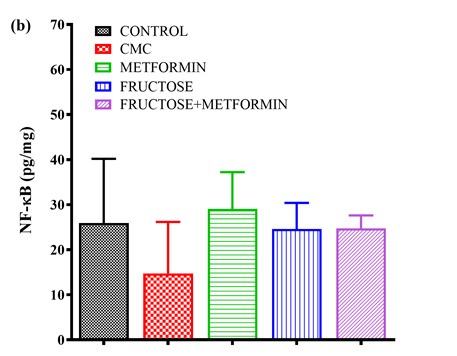


**Fig. S7.** NF-κB levels (pg/mg) measured in ileum tissue of male (a) and female (b) rats (C, n=6; CMC, n=4; M, n=6; F, n=8; FM, n =8), one-way ANOVA, post-hoc Bonferroni test (p>0.05).


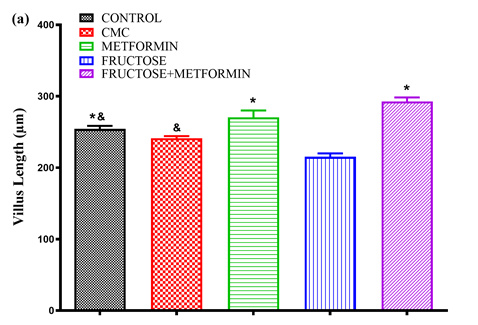

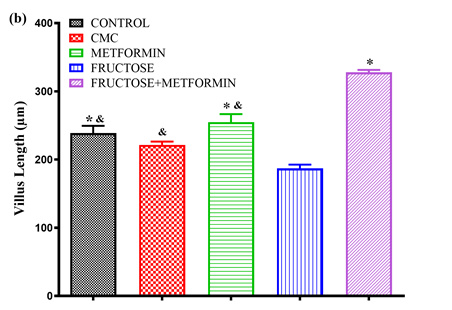


**Fig. S8.** Villus length (µm) measurements of ileum tissue of male (a) and female (b) rats (C, n=6; CMC, n=4; M, n=6; F, n=8; FM, n=8). Intergroup analysis: ^*^Different from F group, ^&^different from FM group, one-way ANOVA, post-hoc Bonferroni test (p<0.05).


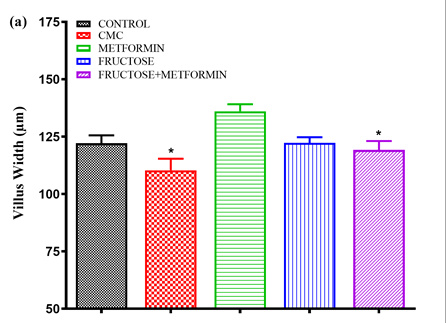

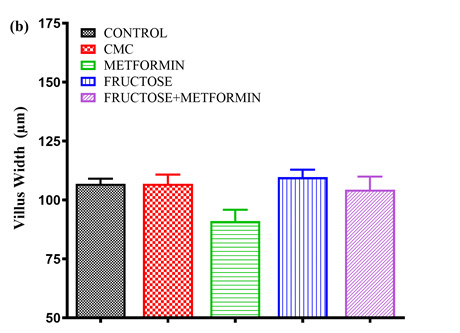


**Fig. S9.** Villus width (µm) measurements of ileum tissue of male (a) and female (b) rats (C, n=6; CMC, n=4; M, n=6; F, n=8; FM, n=8). Intergroup analysis: ^*^Different from M group, one-way ANOVA, post-hoc Bonferroni test (p<0.05).


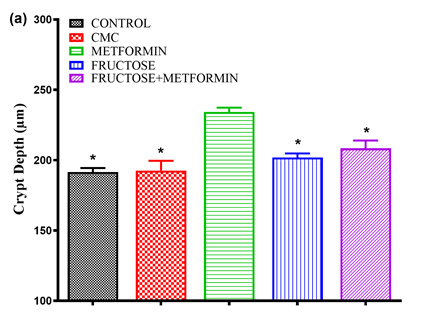

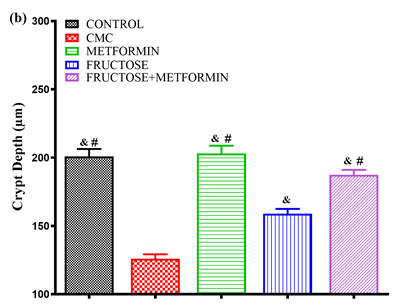


**Fig. S10.** Crypt depth (µm) measurements of ileum tissue of male (a) and female (b) rats (C, n=6; CMC, n=4; M, n=6; F, n=8; FM, n=8). Intergroup analysis: ^*^Different from M group, ^&^different from CMC group, ^#^different from F group, one-way ANOVA, post-hoc Bonferroni test (p<0.05).


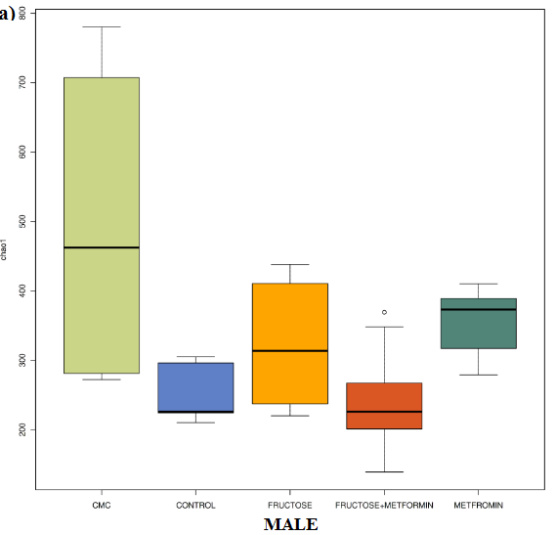

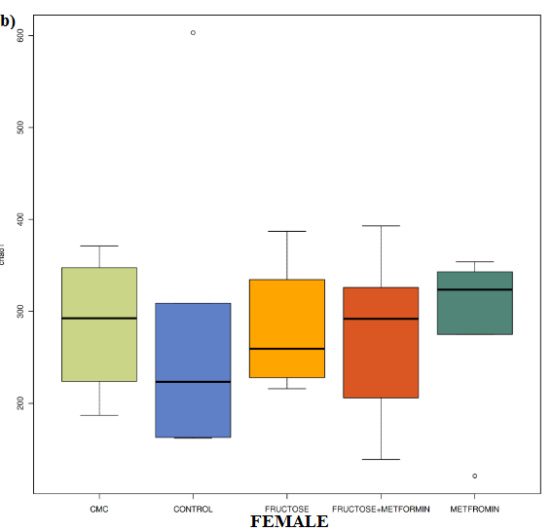

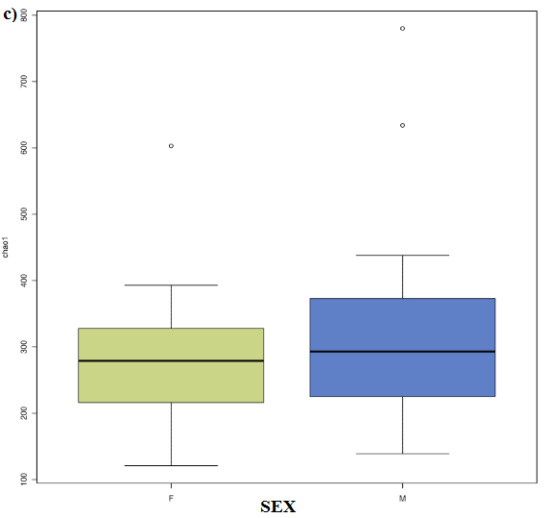


**Fig. S11.** Chao 1 diversity indices of the gut microbiota of male (a), female (b), and male/female (c) rat groups (C, n=6; CMC, n=4; M, n=6; F, n=8; FM, n=8) (Boxes indicate first and third quartiles, dashed lines indicate upper and lower whiskers, and horizontal thick lines indicate median).

**
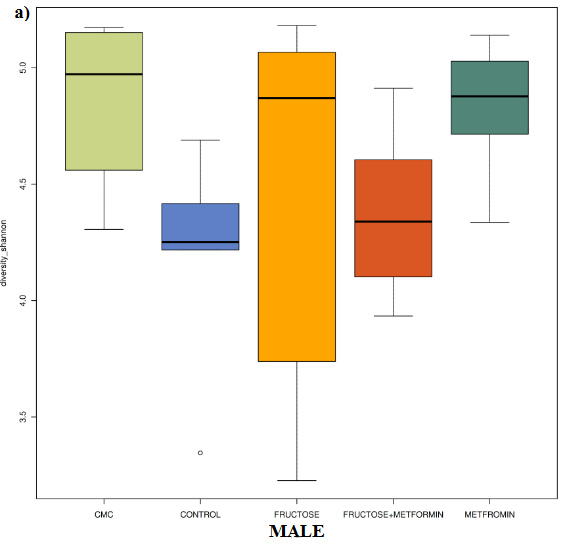

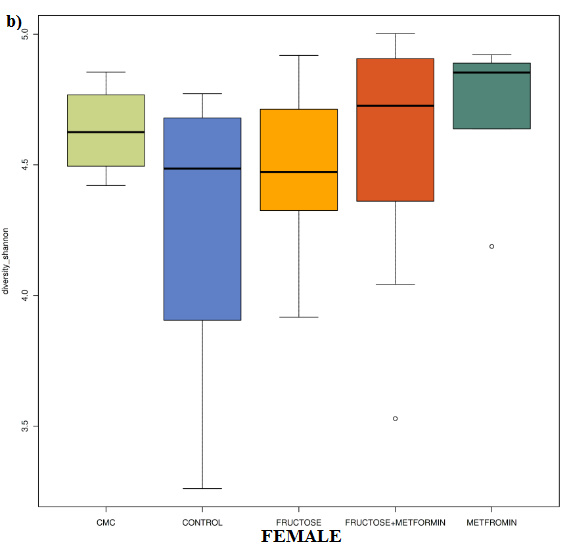
**


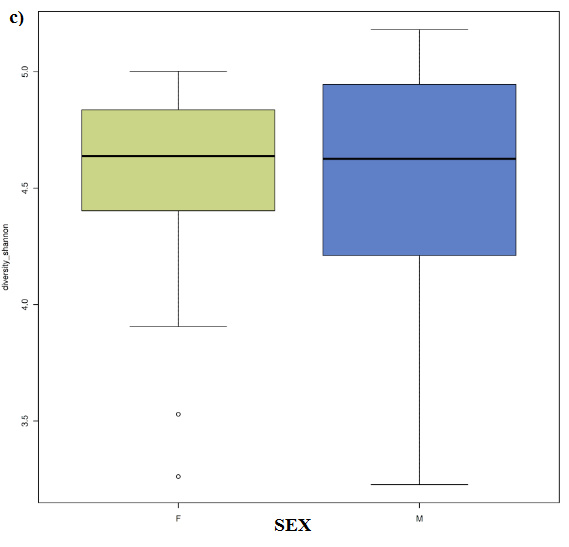


**Fig. S12**. Shannon diversity indices of the gut microbiota of male (a), female (b), and male/female (c) rat groups (C, n=6; CMC, n=4; M, n=6; F, n=8; FM, n=8) (Boxes indicate first and third quartiles, dashed lines indicate upper and lower whiskers, and horizontal thick lines indicate median).


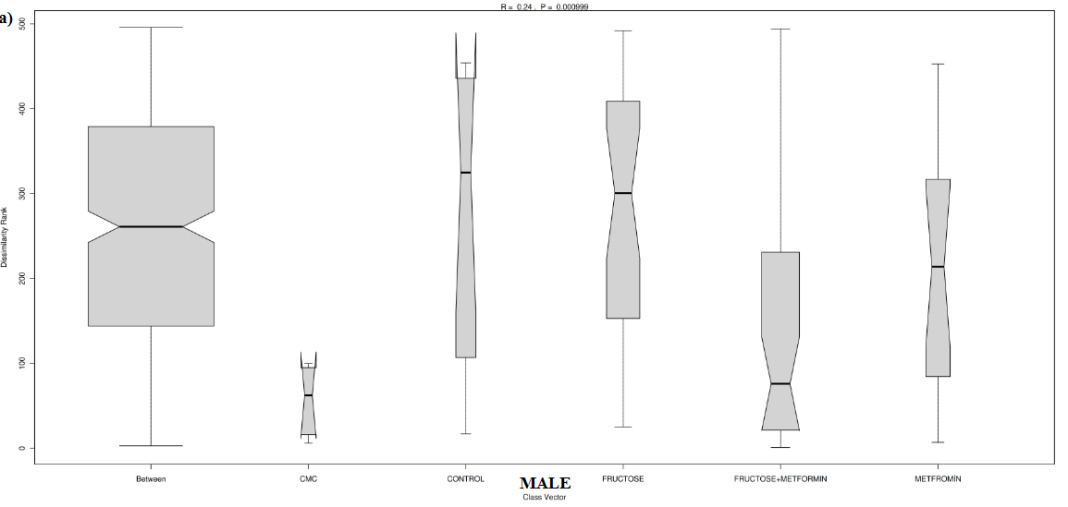

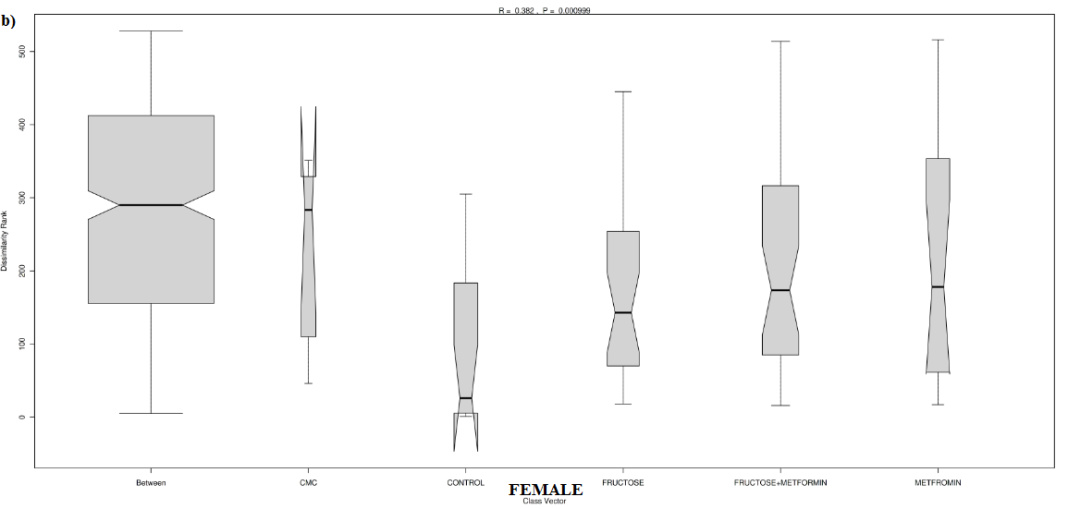

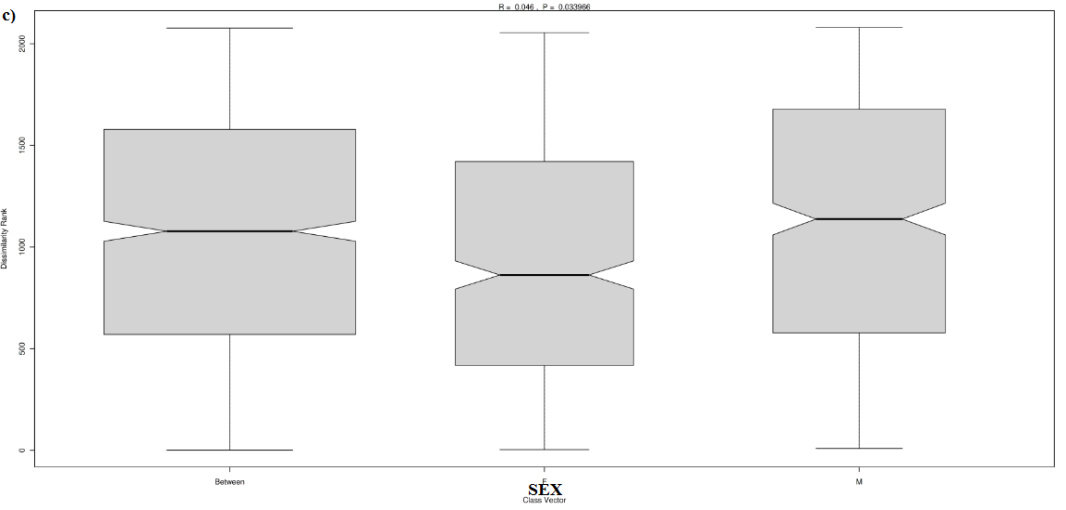


**Fig. S13.** Anosyme analysis of male (a), female (b), and male/female (c) rat groups (C, n=6; CMC, n=4; M, n=6; F, n=8; FM, n=8).
